# Pituitary cell translation and secretory capacities are enhanced cell autonomously by the transcription factor Creb3l2

**DOI:** 10.1038/s41467-019-11894-3

**Published:** 2019-09-03

**Authors:** Konstantin Khetchoumian, Aurélio Balsalobre, Alexandre Mayran, Helen Christian, Valérie Chénard, Julie St-Pierre, Jacques Drouin

**Affiliations:** 1 Laboratoire de génétique moléculaire, Institut de recherches cliniques de Montréal (IRCM), Montréal, QC H2W 1R7 Canada; 2 Departments of Physiology, Anatomy and Genetics, University of Oxford, Oxford, OX2 6HS UK; 3 Department of Biochemistry, McGill University, Rosalind and Morris Goodman Research Centre, Montréal, QC H3A 1A3 Canada

**Keywords:** Transcription factors, Secretion, Translation

## Abstract

Translation is a basic cellular process and its capacity is adapted to cell function. In particular, secretory cells achieve high protein synthesis levels without triggering the protein stress response. It is unknown how and when translation capacity is increased during differentiation. Here, we show that the transcription factor Creb3l2 is a scaling factor for translation capacity in pituitary secretory cells and that it directly binds ~75% of regulatory and effector genes for translation. In parallel with this cell-autonomous mechanism, implementation of the physiological UPR pathway prevents triggering the protein stress response. Knockout mice for *Tpit*, a pituitary differentiation factor, show that Creb3l2 expression and its downstream regulatory network are dependent on *Tpit*. Further, Creb3l2 acts by direct targeting of translation effector genes in parallel with signaling pathways that otherwise regulate protein synthesis. Expression of Creb3l2 may be a useful means to enhance production of therapeutic proteins.

## Abstract (web summary)

Pituitary POMC secreting cells achieve high hormone expression levels after birth but the mechanism for this regulation is unclear. Here, the authors show that this process is driven cell autonomously by the differentiation factor Tpit that activates the bZIP transcription factors Creb3l2 and XBP1 to enhance translation and secretory capacities.

## Introduction

During embryogenesis, specialized cells acquire different size, shape, and organelle content. Some molecular mechanisms at the origin of this morphological diversity were identified in recent years. Indeed, master or scaling transcription factors (TFs), often of the bZIP or bZIP-bHLH type, are associated with biogenesis of endoplasmic reticulum (ER)^1^, Golgi^2–4^, mitochondria^5–7^, lysosomes^8^, or autophagy^9,10^ (reviewed in ref. ^11^). In addition, differentiated cells adapt to different physiological, environmental, or pathological stresses. The best characterized stress response is the ER stress response, also known as unfolded protein response (UPR). In mammalian cells three ER transmembrane proteins—IRE1, PERK, and ATF6 are dealing with ER stress by activating a TF: XBP1s, ATF4, and ATF6N, respectively (reviewed in ref. ^12^). These UPR regulators are the core of a broader family that includes non-canonical UPR regulators^13^, namely the OASIS family of transmembrane bZIP TFs that are ATF6 structural homologs and that have tissue-restricted expression patterns. Their roles remain to be defined in many cases^14^.

Secretory cells, such as pituitary hormone producing cells, have particularly high protein synthesis requirements in the adult where they function as hormone-producing factories. For example, pituitary pro-opiomelamocortin (POMC)-secreting cells (corticotropes of the anterior lobe, AL, and melanotropes of the intermediate lobe, IL) increase their hormone production about 100-fold after birth^15^. The mechanism for this postnatal maturation is unknown. Pituitary hormone production also adapts to physiological or environmental conditions. For example during lactation, prolactin producing cells increase size and expand the Golgi compartment^16^, while in frogs exposed to dark environment, pituitary melanotropes increase secretory capacity and αMSH production to stimulate skin pigmentation^17^. The pituitary thus represents an ideal system to study these mechanisms.

Terminal differentiation of POMC-expressing pituitary cells is triggered by Tpit, a Tbox TF only expressed in these cells^18^. Tpit deficiency results in loss of POMC expression and human *TPIT* mutations cause isolated ACTH deficiency^19,20^. To identify mechanisms of POMC cell adaptation to the heavy biosynthetic burden happening at the fetal-to-adult transition, we use POMC-deficient models to show Tpit-dependent control of translation and secretory capacity through activation of two bZIP TFs, Creb3l2 and XBP1. These TFs exert their cell-autonomous action through direct targeting of genes implicated in translation and ER biogenesis, respectively.

## Results

### Establishment of secretory capacity

**Fig. 1:**
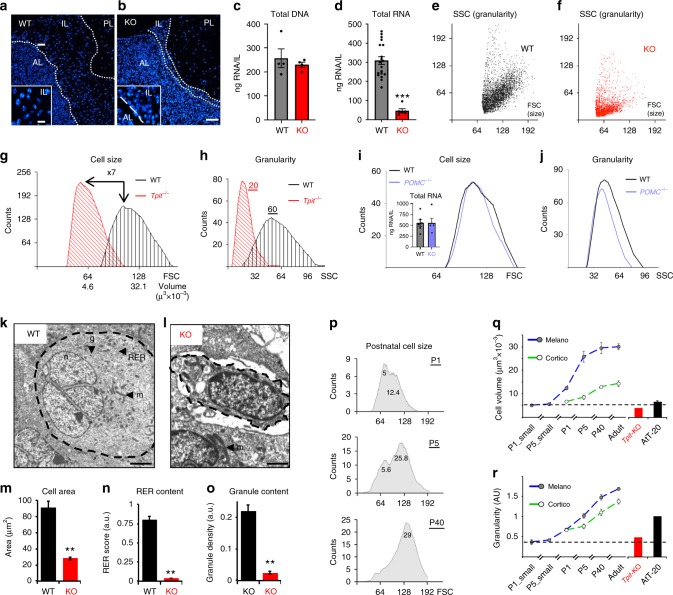
Tpit is required for postnatal maturation of pituitary POMC cells. **a**–**o** Reduced cell size and organelle content in Tpit-deficient pituitaries. **a**, **b** Nuclear staining (Hoechst) of pituitary sections from adult WT **a** and KO **b** mice. Demarcations between pituitary lobes (anterior: AL, intermediate: IL, posterior: PL) are indicated by dashed lines. Higher magnification insets show increased nuclear density in mutant IL. Scale bars: 10 µm **a**, 20 µm **b**. **c**, **d** Quantitation of total genomic DNA **c** and RNA **d** contents in WT and *Tpit* KO IL (each dot represents independent measure). **e**–**h** Flow cytometry (FACS) analysis of WT **e** and *Tpit-KO* **f** IL cells showing forward (FSC) vs. side (SSC) scatter that reflect cell size and granularity (organelle content), respectively. Distribution of cell size **g** and granularity **h** for each genotype. **i**, **j** RNA content (histogram) and FACS analyses of *POMC*-deficient IL cells (purple) indicate normal RNA content, size **i** and slightly reduced granularity **j**. **k**–**o** Ultrastructure analyses confirm cell size and organelle content defects in *Tpit*-deficient IL. **k**, **l** Electron micrographs of sections from WT **k** or KO **l** adult mouse pituitaries. WT cells **k** are rounded (dashed line), contain dense-cored secretory granules (g), mitochondria (m), and some rough endoplasmic reticulum (RER). Mutant cells **l** are smaller, with little cytoplasm and organelles (mitochondria dominate) and have a stellate appearance (not rounded). Scale bar in *k* = 2 µm, in *l* = 1 µm. *n* = nucleus. **m**–**o** Quantitation of cell area **m**, RER content **n** and granule density **o** in WT or KO IL cells. Data are presented as means (*n* = 4 mice) ± SEM. **p**–**r** Pituitary POMC cells develop into secretory factories postnatally. **p** Melanotrope cell size changes after birth. FACS profiles showing FSC distribution of IL GFP-positive cells from *POMC-EGFP* mice. Numbers indicate calculated cell volumes (µm^3^ x 10^−3^). **q**, **r** Summary of size **q** and granularity/organelle content **r** changes in postnatal IL melanotropes (filled circles) and AL corticotropes (empty circles). Inferred progression of cell size and granularity in melanotropes (blue) and corticotropes (green) between days P1 and P90 (adult). Size and granularity of *Tpit-KO* cells remain at the P1 stage (red). Compared to controls using bilateral Student’s *T*-test with unequal variances: *P < 0.05, **P < 0.005, ***P < 0.0005

As marked upregulation of POMC expression is the hallmark of POMC cell postnatal maturation, we first assessed if this process is dependent on differentiation and/or POMC itself. Inactivation of the *Tpit* gene results in loss of POMC expression in both corticotropes and melanotropes^20^. In addition, Tpit-deficient pituitaries show a dramatic reduction of intermediate lobe (IL) size (Fig. 1a, b), suggesting there are either fewer cells or decreased cell size. To test the first hypothesis, total IL DNA content was determined. Wild-type (WT) and *Tpit* knockout (KO) tissues contained the same amount of DNA (Fig. 1c), indicating that cell number is not affected in the absence of Tpit. In contrast, the RNA content of *Tpit* KO IL was reduced 6.6-fold (Fig. 1d). Moreover, IL nuclear staining (Hoechst) showed increased nuclear density in mutant IL (Fig. 1a, b insets), suggesting that Tpit-deficient cells are smaller. FACS analysis confirmed this, and also revealed reduced organelle content (granularity) (Fig. 1e, f). The reduction of *Tpit* KO IL cell volume was found to be seven-fold compared to WT (Fig. 1g), while cell granularity was decreased three-fold (Fig. 1h). Thus, postnatal maturation of cell size and secretory organelle content appears to be Tpit-dependent.

The decrease of cell size and granularity may be secondary to the absence of POMC mRNA in *Tpit* KO cells, since this mRNA constitutes their major translation burden. We used *POMC* KO IL cells to assess this possibility. Strikingly, IL RNA content, cell size, and organelle contents were not affected by the absence of POMC mRNA (Fig. 1i, j). In order to directly ascertain the putative loss of organelles in Tpit-deficient cells, we performed electron microscopy. Whereas WT melanotropes (Fig. 1k) are rounded, contain dense secretory granules, mitochondria, and rough endoplasmic reticulum (RER), KO cells (Fig. 1l) appeared to be smaller, with little cytoplasm or organelles. Quantitation of these features revealed reduced cell area, RER, and granule content (Fig. 1m–o) in KO IL cells. In summary, postnatal maturation of pituitary POMC cells is part of the Tpit-dependent differentiation program and is not secondary to the translational burden of the POMC mRNA.

In addition to the 100-fold increase of POMC mRNA levels in adults^15^, examination of POMC cells suggested that they increase in volume during postnatal development. We took advantage of *POMC-EGFP* reporter mice^15^ to analyze by FACS the time course of this increase. Both melanotropes and corticotropes increase in size between postnatal days P1 and P40, with greater amplitude in melanotropes (Fig. 1p, q). In addition, an increase of cell granularity was observed (Fig. 1r), suggesting an expansion of organelle content. In summary, maturation of POMC cell secretory capacity is implemented during the postnatal period and it is triggered by Tpit.

### Creb3l2 a Tpit-dependent regulator

**Fig. 2:**
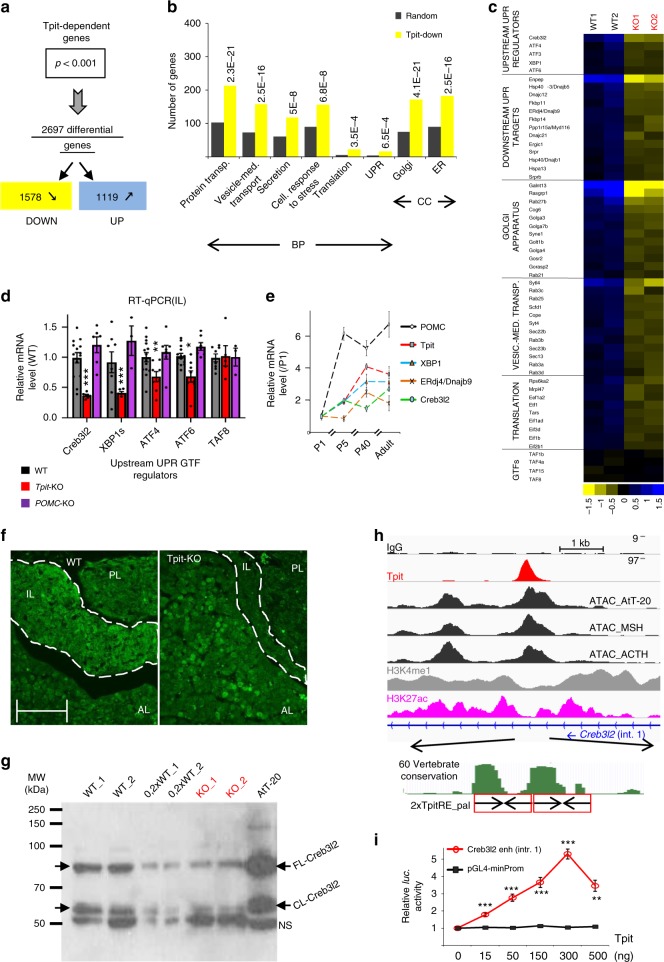
Tpit controls expression of translation and secretory pathway genes. **a**–**e** Tpit transcriptional signature in the pituitary. **a** Tpit-dependent genes identified by comparison of WT and KO IL transcriptomes. **b** Gene ontology (GO) distribution of transcripts downregulated in *Tpit*-deficient IL (yellow compared to random in gray) showing enrichment of protein transport, secretory pathway, and translation genes. BP biological process. CC cellular component. *P*-values relative to random occurrence using modified Fisher Exact *P*-value (EASE scores provided by DAVID) are shown. **c** Heat map representation (log_2_ of changes relative to median) of expression for a subset of genes in two WT and KO ILs. Each column represents an IL sample, and each row a gene. Blue and yellow represent up- and down- regulation, respectively. GTFs general transcription factors. **d** Real-time quantitative PCR (RT-qPCR) validation of expression changes for a subset of UPR pathway genes in *Tpit*-KO (red) and *POMC*-KO ILs (purple). Relative mRNA levels were normalized to TBP mRNA and represented as fraction of WT levels (black). Means ± SEM of 3–6 mice per genotype (each dot represents independent measure). **e** Postnatal expression of *Tpit, POMC, Creb3l2*, and UPR pathway genes in melanotropes. Relative IL mRNA levels (means ± SEM of 5–8 animals per group) measured by RT-qPCR were normalized to TBP and represented as fraction of P1 levels. **f** Loss of Creb3l2 expression in *Tpit* KO IL assessed by Creb3l2 immunofluorescence on P17 pituitary sections. **g** Western blot analysis of Creb3l2 in WT and Tpit KO ILs, and in AtT-20 cells. FL, full-length, CL, cleaved, NS, non specific. **h**, **i** Creb3l2 is a major Tpit target. **h** *Top*: ChIPseq profiles in AtT-20 cells revealing direct recruitment of Tpit to a *Creb3l2* intron 1 sequence exhibiting active enhancer marks, namely bimodal H3K4me1, H3K27ac and ATAC peaks. ATACseq profiles in FACS-purified normal mouse melanotropes (MSH) and corticotropes (ACTH) are shown for comparison. *Bottom*: two conserved Tpit-RE palindromic sequences at the Tpit recruitment region of *Creb3l2* intron 1. **i** Dose-dependent activation by Tpit of *Luciferase* reporter containing the intron1 enhancer (986 bp) of the *Creb3l2* gene assessed by transfection into Tpit-negative αT3 pituitary cells. Compared to controls using bilateral Student’s *T*-test with unequal variances: *P < 0.05, **P < 0.005, ***P < 0.0005

To gain insights into the molecular mechanisms of Tpit-dependent POMC cell maturation, we compared gene expression profiles of WT and *Tpit* KO IL that contain mostly melanotropes^21^. Comparison of WT versus KO gene expression profiles (Supplementary Fig. 1a–c) revealed 2697 differentially expressed transcripts using a *P*-value cut-off *P* < 0.001 (Fig. 2a and Supplementary Data 1). Gene ontology (GO) analysis revealed that the most significantly enriched biological processes (BP) associated with 1578 transcripts downregulated in Tpit-deficient IL are intracellular protein transport, secretory pathway, and translation (Fig. 2b). In particular and as validated by RT-qPCR, major regulators of the UPR pathway—XBP1, ATF4, and ATF6, as well as numerous downstream UPR pathway, vesicle-mediated transport and translation control genes are *Tpit*-dependent (Fig. 2c, d). These genes were not downregulated in *POMC*-KO ILs (Fig. 2d). Hence, expression of Tpit, but not POMC, correlates positively with that of secretory pathway genes. Interestingly, UPR genes associated with translational repression (eIF2α kinase *Perk/Eif2ak3*), apoptosis (*Chop/Ddit3*) or ER-associated protein degradation (ERAD) are not affected by loss of Tpit (Supplementary Fig. 1d). In addition, expression of *Ppp1r15a*/*Gadd34/Myd116* (phosphatase that reverses inhibitory eIF2α phosphorylation) and *Naip5*-*Naip6* (anti-apoptotic genes) is decreased in *Tpit KO* IL. Therefore, Tpit action correlates with activation of some branches of the UPR pathways but not with those involved in translational attenuation, disposal of misfolded proteins, and programmed cell death, that are part of the classical XBP1-dependent UPR stress response. These differences distinguish the physiological UPR from the ER stress-related UPR response.

Of the other bZIP factors affected by the *Tpit* KO, Creb3l2 is most expressed in the pituitary and it is the most downregulated gene of the OASIS subfamily in *Tpit* KO (Fig. 2c, d and Supplementary Fig. 1e, f). Expression of both *Creb3l2* and *XBP1*, the classical UPR responsive TF, follow the Tpit/POMC postnatal expression profile (Fig. 2e), in agreement with the hypothesis that they mediate Tpit actions on postnatal maturation of POMC cells. Consistent with the decrease of *Creb3l2* mRNA, Creb3l2 protein is not detectable by immunohistofluorescence in the IL of *Tpit* KO pituitaries (Fig. 2f). We did Western blot analyses of Creb3l2 in WT and *Tpit* KO ILs in order to more quantitatively assess Creb3l2 levels and processing status (Fig. 2g). Indeed, the OASIS subfamily of TFs are typically activated by proteolytic cleavage to release an active form. Despite showing Creb3l2 levels that are about five times lower in IL KO compared to WT, both samples have similar ratios of cleaved (CL) to full-length (FL) Creb3l2 (Fig. 2g); this ratio is also similar in AtT-20 cells. Similar findings were obtained for transfected Flag-tagged FL-Creb3l2 in AtT-20 cells (Supplementary Fig. 2a) and Creb3l2 levels were increased after treatment with the proteasome inhibitor MG132 as previously shown^22^. The presence of CL-Creb3l2 in unstressed normal pituitary tissue suggests that cleavage is either cell-autonomous or may be responsive to signals as shown for TGFβ-dependent Creb3l2 cleavage in liver^23^.

In order to correlate Tpit-dependence with Tpit action, we queried the Tpit ChIPseq data^24^ for direct binding to putative *Creb3l2* regulatory sequences. Strong Tpit binding (8th highest peak out of 17,190) is present in the first intron of the *Creb3l2* gene (Fig. 2h). This site contains two conserved juxtaposed TpitRE palindromic sequences^25^ and exhibits the chromatin signature (H3K4me1, H3K27ac, and ATACseq peak) of active enhancer sequences; the AtT-20 ATACseq profiles at this locus are similar to those present in normal mouse pituitary melanotropes and corticotropes (Fig. 2h). When directly tested in a luciferase reporter assay, this sequence behaved as a bona fide Tpit-responsive enhancer (Fig. 2i). Altogether, these results identify *Creb3l2* as a major Tpit target gene.

### Translation and secretory capacity

**Fig. 3:**
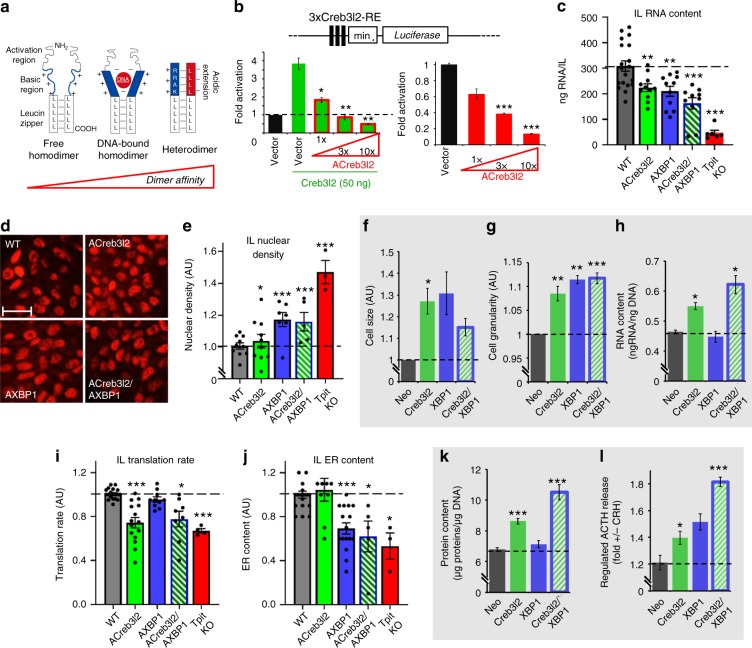
Creb3l2 and XBP1 regulate different aspects of secretory capacity. **a** Schematic view of dominant negative inhibition of bZIP TFs by AZIP proteins (adapted from ref. ^28^). *Left*: free bZIP/bZIP homodimer with unstructured basic region (blue). *Middle*: DNA bound bZIP/bZIP homodimer with α-helical basic region. *Right*: bZIP/AZIP heterodimer where the basic region and the designed acidic amphipathic extension (red) interact as α-helices to extend the coiled-coil domain and prevent DNA binding. **b** Efficiency of overexpressed (*left*) or endogenous (*right*) Creb3l2 inhibition by ACreb3l2. The Creb3l2-RE *Luciferase* reporter (3xCreb3l2-RE-*luc*) was used to assess Creb3l2 activity and ACreb3l2 antagonism upon transfection into INS-1 cells; similar results were obtained in AtT-20 cells. **c** Total IL RNA contents measured in WT, *ACreb3l2*, *AXBP1*, *ACreb3l2/AXBP1* transgenic, and *Tpit-KO* mice. Data are from 6 to 19 pools of 5–10 ILs. **d**, **e** Melanotrope nuclear density (Tpit nuclear staining: red) on histological sections of ILs from WT, *ACreb3l2*, *AXBP1*, and *ACreb3l2/AXBP1* transgenic mice **d** and their quantifications in arbitrary units (AU) relative to WT melanotrope density adjusted to 1 **e**. **f**–**h** Creb3l2 and XBP1 gain of function in stably infected AtT-20 cells. FACS analyses showing cell size **f** and granularity **g** index for AtT-20 cells expressing Creb3l2, XBP1, or both compared to control neomycin-resistant (Neo) AtT-20 cells. Data (±SEM, *n* = 3) are presented relative to Neo cells set to 1. **h** Total RNA content of control AtT-20 cells (Neo) and AtT-20 cells expressing Creb3l2, XBP1 or both (±SEM, *n* = 3). **i**–**l** Relative contributions of Creb3l2 and XBP1 to secretory capacity. **i** SUnSET (puromycin incorporation) measurements of protein synthesis in IL cells of control (WT), *ACreb3l2*, *AXBP1*, *ACreb3l2/AXBP1* transgenic, and *Tpit-KO* mice. Data in AU are presented as fractions of WT IL translation rates set to 1. **j** Melanotrope cell ER contents (ER-tracker) measured in WT, *ACreb3l2*, *AXBP1*, *ACreb3l2/AXBP1* transgenic, and *Tpit-KO* mice. Data are presented as fraction of WT IL ER contents set to 1. **k** Total protein content of control AtT-20 cells (Neo) and AtT-20 cells expressing Creb3l2, XBP1, or both. **l** CRH-induced ACTH release from the indicated pools of AtT-20 cells. Each dot represents independent measure. Compared to controls using bilateral Student’s *T*-test with unequal variances: * *P* *<* 0.05, **P *<* 0.005, ***P *<* 0.0005

In order to evaluate in vivo the putative roles of Creb3l2 and XBP1, we performed loss-of-function and gain-of-function (LOF, GOF) experiments. Because Creb3l2 or XBP1 germline inactivation lead to perinatal or embryonic lethality^26,27^, we used a pituitary POMC cell-specific dominant-negative (DN) approach. Replacement of the basic region of bZIP proteins by an acidic (A) sequence (AZIP) was shown to be a very efficient and specific way to inactivate endogenous bZIP factors^28^ (Fig. 3a). We generated ACreb3l2 and AXBP1 constructs and validated their efficiency and specificity using a 3xCreb3l2-RE-luc reporter in both activated and basal conditions (Fig. 3b and Supplementary Fig. 2b, c). To inhibit Creb3l2 or XBP1 in pituitary POMC cells, we expressed ACreb3l2 or AXBP1 in transgenic mice using the POMC promoter, previously shown to be specific and penetrant in melanotrope cells^15^. For each transgene, we selected two stable mouse lines with an AZIP versus endogenous bZIP mRNA ratios > 5, ensuring strong inhibition of the endogenous TF (Fig. 3b and Supplementary Fig. 2b–e). Both transgenic lines behaved similarly and results are shown for one line. Total RNA content of transgenic ILs was reduced with synergistic effects in double ACreb3l2/AXBP1 transgenic mice (Fig. 3c); as total RNA mostly reflects rRNA content, we confirmed by RT-qPCR that these changes are essentially due to similar changes in rRNA levels (Supplementary Fig. 2f). Also, ACreb3l2 and AXBP1 increased IL nuclear density, reflecting a reduction in melanotrope cell size (Fig. 3d, e).

To complement these in vivo LOF models, we assessed the consequences of Creb3l2 or/and XBP1 overexpression (GOF) on size and secretory capacity of POMC cells by generating stable populations of AtT-20 cells that overexpress active forms of Creb3l2 (cleaved Creb3l2) and/or XBP1 (spliced XBP1). Expression of the active forms of Creb3l2, XBP1, or both led to a similar increase of AtT-20 cell size and granularity (Fig. 3f, g). In contrast, Creb3l2, but not XBP1, overexpression resulted in higher RNA contents, and this effect is even greater in Creb3l2 + XBP1 overexpressing cells (Fig. 3h). Hence, Creb3l2 and XBP1 are controlling overlapping (cell size and organelles) and unique (RNA content) aspects of secretory capacity.

As the *Tpit*-dependent transcriptome (Fig. 2b, c) identified translation and ER biogenesis, we assessed these functions in the in vivo LOF and AtT-20 GOF models. Strikingly, Creb3l2, but not XBP1, inhibition led to a specific reduction of translation rates (Fig. 3i), as assessed by the puromycin incorporation assay^29^. This effect fully mimics the loss of *Tpit*. There is an apparent discrepancy between RNA content (Fig. 3c) and translation rates (Fig. 3i). This could be due to the brief assessment of nascent polypeptide synthesis measured by the puromycin assay compared to the cumulative effect on RNA accumulation. In contrast, ER content is dependent on XBP1, but not Creb3l2 (Fig. 3j), and XBP1 inhibition also mimics the *Tpit* KO phenotype. Altogether, these results show that Creb3l2 and XBP1 have different activities, namely regulation of translation and ER biogenesis, respectively. The association of XBP1 with ER biogenesis is entirely consistent with prior work in other systems^1,30,31^ and thus provides validation for the specificity of our DN approach. Further, we validated our association of Creb3l2 with translation by reanalyzing the transcriptome data (Supplementary Fig. 2g) from a recently published study on pharmacological inhibition of Creb3l2 processing in B cells^32^. Consistent with the role of Creb3l2 in translation, Creb3l2, but not XBP1 overexpressing cells have higher total protein content (Fig. 3k). An even greater increase was observed in Creb3l2 + XBP1 overexpressing cells.

We also assessed the impact of Creb3l2 and XBP1 on the regulated secretory pathway^33^ by measuring ACTH release in response to corticotropin-releasing hormone (CRH). Both, Creb3l2 and XBP1 overexpression improved CRH response, with a synergistic effect in Creb3l2 + XBP1 cells (Fig. 3l). Collectively, these results suggest that Creb3l2 and XBP1 are jointly regulating the development of secretory function.

The Akt/ mTOR pathway regulates translation in response to growth factors; in order to assess whether this pathway is involved in the action of Creb3l2, we measured by Western blot the levels of phospho-mTOR and mTOR. These are largely unaltered by *Tpit* KO, the *ACreb3l2* or *AXBP1* transgenes (Supplementary Fig. 2h) suggesting that Creb3l2 may act downstream or in parallel with this pathway.

### Creb3l2 targets the promoters of translation genes

**Fig. 4:**
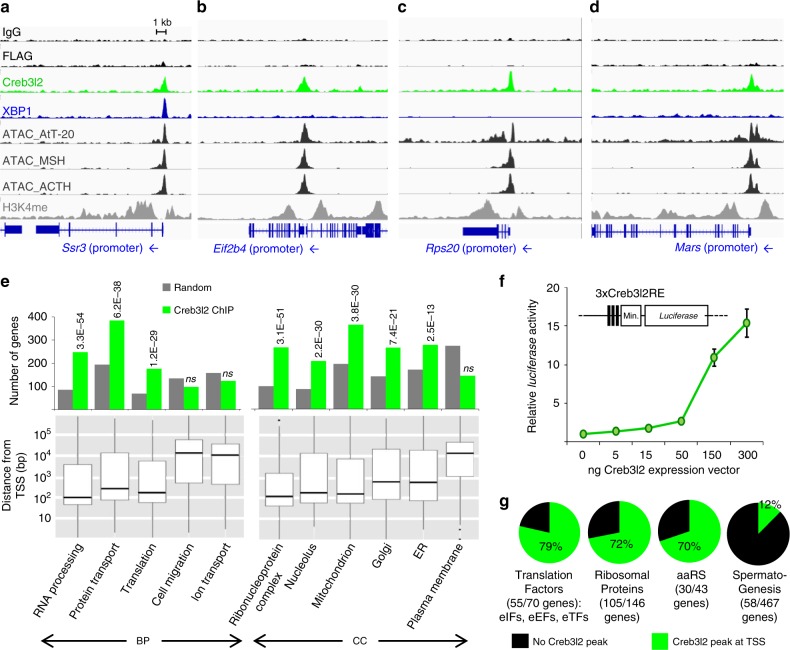
Creb3l2 targets the promoters of translation genes. **a**–**d** ChIPseq profiles for Creb3l2 and XBP1 at regulatory sequences of ER **a** and translation **b**–**d** genes. ChIPseq patterns are shown for control IgG and FLAG, Creb3l2, XBP1, H3K4me1, and ATACseq. ATACseq profiles in FACS-purified normal mouse melanotropes (MSH) and corticotropes (ACTH) are shown for comparison. **e** GO terms of genes associated with TSS proximal (<1 kb) Creb3l2 peaks. Peaks were assigned to the closest gene with the AnnotatePeaks Homer command. Bars represent the number of genes in each category associated with Creb3l2 peaks (green) or random occurrence of genes in each category (gray). *P*-values relative to random occurrence using hypergeometric distribution provided by AmiGO are shown above the bars. ns not significant. The bottom panels provide boxplot representation of the distance to TSS for Creb3l2 peaks of each GO category revealing promoter-proximal associations for groups with significant associations. Center lines show medians; box limits indicate the 25th and 75th percentiles; whiskers extend to 1.5 times the interquartile range from the 25th to 75th percentiles. **f** Dose-dependent activation by Creb3l2 of luciferase reporter containing three copies of Creb3l2-binding sites (3xCreb3l2-RE) upon transfection into pituitary GH3 cells. **g** Creb3l2 binds 70–80% of the promoters of translation genes. Pie charts showing proportion of genes (absolute gene numbers indicated between parentheses and target genes listed in Supplementary Table 2) bound by Creb3l2 (green) in each category related to translation, namely: translation factors, ribosomal proteins, and aminoacyl tRNA synthetases. The spermatogenesis group serves as control. Gene lists for each category are from MGI (http://www.informatics.jax.org/mgihome/GO/project.shtml)

In order to define the mechanism by which Creb3l2 may enhance translation capacity and to document XBP1 action on the physiological UPR pathway, we performed ChIPseq for both factors in AtT-20 cells. We observed 6484 Creb3l2 recruitment peaks, while XBP1 ChIPseq produced fewer peaks (258) similar to observations made in other cells^34,35^. Contrary to many TFs such as Tpit that are mostly recruited at enhancers, 41% of Creb3l2 and XBP1 peaks are found at promoters (Supplementary Fig. 3a–c). The reliability of these ChIPseq data was validated by ChIP-qPCR of representative loci (Supplementary Fig. 3d, e, f). While XBP1 targets genes involved in ER biogenesis (Supplementary Fig. 3g) in agreement with previous reports^34^, Creb3l2 peaks are enriched at regulatory sequences of genes controlling translation (Fig. 4a–e and Supplementary Fig. 3h–k). The bulk of Creb3l2 and XBP1 peaks are associated with active chromatin marks, namely DNA accessibility revealed by ATACseq and methylated H3K4me (Supplementary Fig. 3l, m).

GO analysis of genes associated with Creb3l2 peaks confirmed enrichment of categories related to translation, protein transport, and RNA processing (Fig. 4e, upper panel), while XBP1-associated genes correspond to ER stress/UPR (Supplementary Fig. 3g, upper panel). These associations mostly occur at promoters/proximal regions of target genes (Fig. 4e and Supplementary Fig. 3g, bottom panels). Collectively, these data show targeting of two subsets of genes involved in secretory function, namely genes involved in translation and protein transport (controlled by Creb3l2) and ER biogenesis (controlled by XBP1).

Analysis of the ChIPseq data for de novo motifs revealed conserved sequence motifs for Creb3l2 and XBP1 (Supplementary Fig. 4a, b) that are consistent with prior work^24,25,34,36^. Creb3l2 and XBP1 peaks were associated with bZIP motifs containing the core ACGT sequence. A reporter containing three copies of the Creb3l2-RE upstream of a minimal promoter, as well as a natural promoter (*Ssr3*) and enhancer (*Kcnma1*) were activated dose-dependently by both Creb3l2 and XBP1 (Fig. 4f and Supplementary Fig. 4c, d). Thus, Creb3l2 and/or XBP1 directly activate genes involved in translation and secretory system development.

In order to better assess the extent of Creb3l2 targeting of genes involved in translation, we computed the number of genes encoding translation initiation (eIF), elongation (eEF), and termination (eTF) factors, as well as ribosomal protein-coding genes and those for aminoacyl tRNA synthethases that are directly targeted by Creb3l2 binding (within 100 bp of their TSS) based on the ChIPseq data. This showed direct targeting of 70–80% of these genes depending of the sub-categories (Fig. 4g, Supplementary Data 2). Collectively, these data indicate that Creb3l2 is acting very broadly on a large number of translation regulatory and effector genes in order to enhance translation capacity. This situates the Creb3l2 cell-autonomous action in parrallel with the signaling pathways that are usually implicated in translational control, such as the Akt/mTOR pathway.

### Creb3l2 a translation scaling factor

**Fig. 5:**
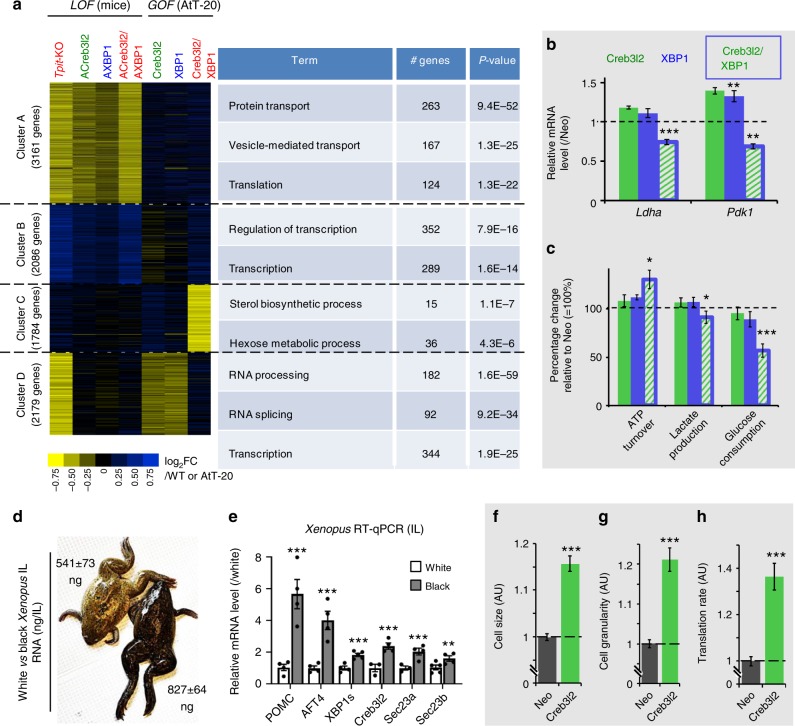
Creb3l2 is a scaling factor for translation. **a** *Tpit* KO transcriptome signature is largely phenocopied by inhibition of *Creb3l2* and *XBP1*. Heatmap representation of gene clustering identified by global analysis of expression profiling datasets (*left*) and GO distribution of genes in each cluster (*right*). Transcriptome data from WT, *Tpit-KO*, *ACreb3l2*, *AXBP1*, and *ACreb3l2/AXBP1* transgenic mice and from the gain-of-function AtT-20 cells were clustered with the Cluster 3.0 software using *K*-means. *P*-values relative to random occurrence using modified Fisher Exact *P*-value (EASE scores provided by DAVID) are shown. **b** Decreased lactate dehydrogenase (Ldha) and pyruvate dehydrogenase kinase (Pdk1) mRNAs (RNAseq normalized counts) in AtT-20 cells expressing Creb3l2 + XBP1 relative to control (Neo) cells set to 1. **c** Increased ATP turnover, decreased lactate production and glucose utilization, showing shift of ATP production from glycolysis to oxidative phosphorylation in AtT-20 cells expressing Creb3l2 + XBP1. Data represent the means ± SEM (*n* = 5–6). **d** Adaptation (5 weeks) of *Xenopus* skin color to black (*right*) background compared to white (*left*) increases total IL RNA content (ng ± SEM, for *n* = 4–5). **e** Expression of *POMC* and UPR genes in white (white bars) and black (black bars) background adapted frogs. Relative IL mRNA levels measured by RT-qPCR were normalized to those of β*-actin* and represented as fraction of levels in white background set to 1. Data represent means ± SEM (each dot represents independent measure). **f**–**h** Expression of Creb3l2 in INS-1 cells. FACS analyses of cell size **f** and granularity **g** index for INS-1 cells expressing Creb3l2. All data represent the means ± SEM (*n* = 3). **h** SUnSET measurement of protein synthesis in control INS-1 cells (Neo) and INS-1 cells expressing Creb3l2. Data (±SEM, *n* = 3) are presented relative to Neo cell set to 1. Compared to controls using bilateral Student’s *T*-test with unequal variances: *P *<* 0.05, **P < 0.005, ***P < 0.0005

To evaluate to which extent gene expression changes dependent on Creb3l2 or/and XBP1 resemble gene expression changes caused by *Tpit* inactivation, we performed RNAseq on our LOF and GOF models and analyzed the results by unsupervised clustering analysis (RT-qPCR validation of representative genes is provided in Supplementary Fig. 1f). Four gene clusters (A–D) were identified and analyzed by gene ontology (GO) (Fig. 5a). Two clusters appeared to be particularly interesting: clusters A and C. Cluster A is enriched in genes controlling protein transport and translation that tend to be downregulated in *Tpit* KO tissues. The same tendency is observed for these genes in ACreb3l2 and AXBP1 transgenic mice, and even more in combined ACreb3l2/AXBP1 mice. Cluster A includes about 50% of the genes in each translation sub-category (Fig. 4g). Hence, combined downregulation of Creb3l2 and XBP1 phenocopies the effect of *Tpit* deficiency on translation and secretory pathway genes.

Cluster C represents a group of genes synergistically downregulated in Creb3l2 + XBP1 cells and it is enriched in genes controlling hexose metabolism. Their analysis suggested a shift from glycolysis to oxidative phosphorylation utilization of glucose and in agreement with this, mRNA levels of lactate dehydrogenase (Ldha) and pyruvate dehydrogenase kinase (Pdk1) are decreased in Creb3l2 + XBP1 cells (Fig. 5b). Accordingly, the latter cells exhibit decreased lactate production and glucose utilization (Fig. 5c). We directly assessed cell respiration and found increased ATP turnover only in Creb3l2 + XBP1 cells (Fig. 5c). Since AtT-20 cells are tumor-derived, they are likely to exhibit a Warburg shift towards glycolytic glucose utilization compared to normal pituitary cells. The Creb3l2/XBP1-dependent reverse shift towards the more energy-efficient mitochondrial oxidative phosphorylation pathway is consistent with the high-energy requirements for translation and the development of a high capacity secretory system.

In order to assess conservation of Creb3l2 and UPR regulators in an evolutionary distant but related system, we queried their status in the *Xenopus* IL, where melanotrope cell size and αMSH secretion increase when frogs adapt to a dark background environment^17,37^. We found that IL RNA content (Fig. 5d) and expression levels of UPR pathway regulators, including Creb3l2, are increased in dark-adapted frogs (Fig. 5e). Thus, the physiological UPR pathway appears an evolutionary conserved mechanism to match secretory capacity with translational burden.

To our knowledge, Creb3l2 is the first TF shown to directly target and regulate the translation transcriptome. Creb3l2 is expressed in many secretory tissues (Supplementary Fig. 1f) including in pancreatic islets. While pituitary POMC cell maturation is regulated by Tpit, pancreatic β cell maturation is dependent on the TF Pdx1. Interestingly, Pdx1 binding is observed at the *Creb3l2* promoter in islet cells (Supplementary Fig. 4e) suggesting a maturation mechanism that may resemble that of POMC cells. To assess Creb3l2 activity in β cells, we stably overexpressed Creb3l2 in insulin-secreting INS-1 cells and found an increase of INS-1 cell size, granularity, and a 36% increase of translational rate (Fig. 5f–h). Thus, Creb3l2 appears to be a general regulator of translation capacity.

## Discussion

The present work identifies a master TF for control of translation capacity, Creb3l2. Genome-wide approaches demonstrate that Creb3l2 is directly targeting and stimulating transcription of hundreds of genes involved in different steps of translational control, namely 55 translation factors, 105 ribosomal proteins, and 30 aminoacyl tRNA synthetases genes. Genetic manipulations (LOF, GOF) of the *Creb3l2* gene in mice and two different secretory cell types led to significant changes of translation rates (25–36%). Overexpression of Creb3l2 was sufficient to increase overall cellular protein content by ≈25%. Scaling factors were proposed to be factors that adjust expression of genes controlling basic cell functions to specific cellular needs^38^. Creb3l2 is thus a scaling factor for translation.

It is noteworthy that total RNA content is increased following Creb3l2 action. Although we could not find evidence of direct Creb3l2 binding to rRNA genes, its broad action on expression of ribosomal proteins is likely sufficient to increase rRNA transcription and hence, have a significant effect on total RNA content. It was indeed shown that depletion of ribosomal proteins RPS19, RPS6, or RPL11 is sufficient to decrease rRNA transcription^39^.

**Fig. 6:**
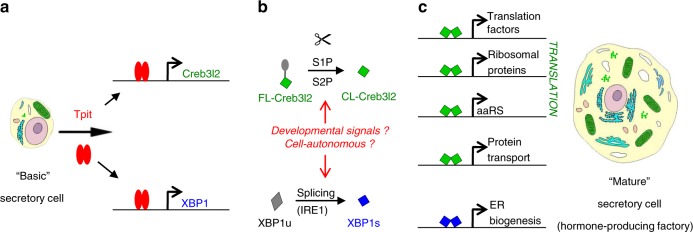
Developmental mechanism to establish high capacity secretory cells. Freshly differentiated pituitary cells have basic secretory features and acquire the features of hormone-producing factories during early post-natal development under the concerted actions of two bZIP transcription factors Creb3l2 and XBP1. This is controlled by the determination transcription factor Tpit in pituitary POMC cells (likely PDX1 in pancreatic β cells and BLIMP1 in activated B cells). **a** In pituitary, Tpit activates expression of *Creb3l2* and *XBP1* by directly targeting their genes. **b** Both factors require activation through either proteolytic cleavage (Creb3l2) or alternative splicing (XBP1) and this appears to be either cell-autonomous in normal development or may be regulated by developmental signals as shown for TGFβ in liver^23^. **c** Translation capacity and protein transport are enhanced by the scaling action of Creb3l2 directly targeting the promoters of a large number of genes involved in translation including genes for regulatory factors, ribosomal proteins, and tRNA aminoacyl transferases. In parallel, XBP1 stimulates ER biogenesis without triggering the protein stress response, thus activating the physiological UPR response

The present work suggests a general paradigm that involves three TFs for maturation of pituitary secretory cells into hormone-producing factories (Fig. 6). A tissue-specific terminal differentiation factor (Tpit in pituitary POMC cells, Pdx1 in pancreatic islet cells) that upregulates two bZIP TFs, Creb3l2 for scaling up translation capacity and XBP1 to increase secretory organellogenesis without triggering the stress response. These physiological regulators of secretory capacity could be useful to increase yields of therapeutic proteins, such as cytokines and monoclonal antibodies that are produced by expression in mammalian cells.

The involvement of the UPR regulator XBP1 in this regulatory network clearly fits within the context of the so-called “physiological UPR” system rather than within the cytoprotective homeostatic response to protein stress. The physiological UPR was described in professional secretory cells, such as B lymphocytes, digestive enzyme-secreting zymogenic cells, salivary glands, exocrine pancreas, or pancreatic beta cells^1,30,31,40^. This proactive UPR engagement is preparing mature secretory cells to massive protein synthesis and secretion, rather than being a response to high secretory load. The existence of two types of UPR (i.e. stress related and physiological) raises the question of how context-dependent UPR specificity is achieved. The stress-related UPR involves three sub-pathways characterized by different ER transmembrane sensor proteins, IRE1, PERK, and ATF6. A clearly undesirable aspect of UPR in highly secreting cells is the PERK branch that leads to global translational repression by phosphorylation and inhibition of eIF2α^41^. Interestingly, PERK expression is barely affected by loss of *Tpit* but expression of Gadd34/Myd116, a phosphatase reversing eIF2α phosphorylation is positively regulated by *Tpit*. In fact, Tpit action not only seems to exclude PERK, but also prepare POMC cells to neutralize PERK activation. Also, genes related with ER-associated protein degradation (ERAD genes) are mostly unaffected in *Tpit-/-* pituitaries. Another undesirable UPR effect in physiological conditions is the apoptotic response that follows prolonged ER stress (reviewed in ref. ^42^). In *Tpit-/-* IL, the expression of CHOP/Ddit3, a major mediator of ER stress-induced apoptosis is not affected. During ER stress, apoptosis can be delayed through expression of inhibitors of apoptosis (IAPs) proteins^43–45^. Expression of two IAPs (Naip5 and Naip6) is strongly dependent on *Tpit* and Tpit binding is observed at these genes (Supplementary Fig. 1d). Thus, Tpit protects POMC cells from apoptotic signals. Collectively, these results suggest that Tpit sets up an efficient secretory system by activating useful (XBP1, Creb3l2) but not detrimental (PERK, CHOP/Ddit3) UPR regulators and stimulating inhibitors of deleterious UPR effects (the eIF2α phosphatase Gadd34/Myd116 and inhibitors of apoptosis Naip5 and Naip6).

Importantly, in addition to XBP1 stimulation of ER biogenesis, XBP1 has a synergistic effect on protein synthesis in combination with Creb3l2, increasing the overall cellular protein content by ≈50% with resulting enhancement of regulated secretion. Translation is the most energy consuming (about 75%) cellular process^46^ and uncontrolled protein synthesis leads to apoptosis^47^; in the latter work on translational activation through the PERK pathway mediated by ATF4 and CHOP^47^, it is noteworthy that the group of stimulated translation control genes included primarily tRNA aminoacylation in contrast to the broad set identified in the present work. Remarkably, the combined Creb3l2 plus XBP1 expression in the tumor-derived AtT-20 cells led to a shift of energy metabolism from glycolysis to oxidative phosphorylation, resulting in more efficient ATP production and providing for support of heavy protein synthesis.

In summary, the present work identified Creb3l2 as a scaling factor for translation and showed that its joint action with XBP1 dramatically increases protein synthesis and secretory capacity (Fig. 6). This regulatory network is triggered by Tpit (Fig. 6a) and likely involves cell-autonomous activation of Creb3l2 and XBP1 (Fig. 6b). The targets of Creb3l2 and XBP1 action are revealing of their purpose. By targeting genes that are downstream within the Creb3l2 regulatory network, Creb3l2 sets the cell-autonomous rheostat for translation capacity, in this instance setting high level capacity. And the joint action of XBP1 on the physiological UPR pathway matches this with increased secretory capacity (Fig. 6c). This reset of protein secretion capacity is separate from, and therefore compatible with, upstream signaling pathways regulating translation, such as the Akt/mTOR pathway.

## Methods

### Animals and genotyping

The generation and genotyping of *Tpit* KO (BALB/c) and POMC-EGFP transgenic (C57BL/6) mice was described elsewhere^15,20^. *POMC*-KO (C57BL/6) mice^48^ were purchased from Jackson laboratory (B6.129 × 1-Pomctm2Ute/J strain, stock number 008115). Young-adult (6 months old) *Xenopus laevis* were kindly provided by Dr. Marko Horb. Frogs were fully adapted to black or white background (6 animals per condition) for 5 weeks. After adaptation, animals were anesthetized by submersion in a solution of benzocaine (0.05%), rapidly decapitated, and their IL dissected, frozen in liquid nitrogen and kept at −80 °C until RNA extraction.

For transgenesis, DNA fragments containing ACreb3l2 or AXBP1 DN cDNA constructs under the control of rat *POMC* promoter and followed by a simian virus 40 (SV40) intron and polyadenylation sequences were microinjected and founder lines were derived as previously described^15^.

All animal experimentation was approved by the IRCM Animal Ethics Committee (Protocol 2017-14) in accordance with Canadian regulations.

### Histology and electron microscopy

Processing of PFA-fixed, paraffin-embedded pituitaries, and immunohistofluorescence were done as described^49^. Immunofluorescence labeling with anti-Creb3l2 (Novus Biologicals, NBP1-88697, dilution 1/30) was performed on paraffin sections^49^. For electron microscopy, adult (4–6-month old) mice were first perfused with PBS without potassium (10 ml/mice at 2 ml/min), then with 2.5% glutaraldehyde (20 ml/mice at 2 ml/min). Pituitaries were then dissected and post-fixed in 2.5% glutaraldehyde for 2 h at +4 °C. For electron microscopy^50^, tissues were contrasted with uranyl acetate [2% (w/v) in distilled water], dehydrated in ethanol and embedded in LR Gold resin (Agar Scientific, London, UK). Ultrathin sections (50–80 nm) were prepared using a Reichart–Jung ultracut microtome, mounted on nickel grids (Agar Scientific, Stanstead, Essex, UK) and examined on a JOEL 1010 transmission electron microscope (JOEL USA Inc., Peabody, MA, USA).

For analysis of cell morphology 10 micrographs of intermediate lobe cells per animal (*n* = 4 mice per group) were taken at a magnification of ×4000 and scanned into Adobe Photoshop (version 5.5) and analyzed using Axiovision (version 4.5) image analysis software. The analyst was blind to the sample code. The following parameters were calculated: cytoplasmic, nuclear, and total cell areas; granule area, granule density, and granule diameter. For measurement of the cell and nuclear areas, margins were drawn around the cell or nucleus respectively and the area was calculated. Cytoplasmic area was determined by substracting nuclear area from total cell area. Granule density was calculated by dividing total granule area by cytoplasmic area. Expansion of the RER and Golgi apparatus was assessed visually and graded on a scale of 0–4 (0, no expansion; 4 the most expansion). These estimates do not provide absolute measurements but do provide a basis for comparison.

### IL cell (melanotrope) analyses

For DNA content measurements, ILs were first digested in 1.5 ml tubes with proteinase K (PK: 30 µg) in 400 µl of PK-lysis buffer (0.5% SDS–100 mM NaCl, 50 mM Tris, pH 7.5–1 mM EDTA) at 55 °C, overnight. After a brief RNase treatment (15 min), ILs were redigested with PK (20 µg, 55 °C, 1 h). Genomic DNA was then precipitated with 1 volume of 4 M ammonium acetate—0.6 volume isopropanol, washed with 70% ethanol and dissolved in 20 µl of 10 mM Tris, pH 8.0 at 50 °C, overnight. DNA was quantitated using TKO100 Fluorometer, following manufacturer’s operating instructions (Hoefer Scientific Instruments) in order to specifically quantify double-stranded nucleic acids. Similar results were obtained for male and female pituitaries.

For RNA content measurements, total RNA from pools of 5 to 10 ILs was extracted using RNeasy Plus Mini kit (Qiagen, 74134) following manufacturer’s recommendations, including the step of genomic DNA removal. Experiments were repeated on pools of 5–10 ILs at least six times. Statistical significance was assessed using bilateral Student’s *T*-test with unequal variances on Microsoft Excel. Similar results were obtained for male and female pituitaries.

Translation monitoring was done using the principles of surface sensing of translation (SUnSET)^29^ adapted for in vivo studies^51^. Briefly, adult (3–6 months female) mice were intraperitoneally injected with 100 µl of 7 mg/ml puromycin dihydrochloride (Gibco A1113803) solution (0.04 µmol/kg). 30 min after injection ILs were extracted, and melanotrope cells prepared as described^52^. After fixation and permeabilization (BD Cytofix/Cytoperm 554714) cells were stained in 96-well plates with anti-Puromycin Alexa647 antibodies (mouse monoclonal, Millipore MABE343-AF647, dilution1/75) in 30 µl of 1xPBS–0.5% BSA (+4 °C, 30 min). Cells were then washed in 1xPBS–0.1% BSA, resuspended in washing buffer and analyzed by FACS. The experiment was repeated on pools of 4–6 ILs at least five times. Statistical significance was assessed using bilateral Student’s *T*-test with unequal variances on Microsoft Excel.

For ER content measurements, melanotrope cells were prepared as described above. Living cells were then stained with 1 µM ER-tracker green (ThermoFisher Scientific E34251) in 30 µl HBSS (96-well plates) at 37 °C, 5% CO_2_. Cells were then washed in 1xPBS–0.1% BSA, resuspended in the same washing buffer and analyzed by FACS. The experiment was repeated on pools of 3–5 IL at least three times. Statistical significance was assessed using bilateral Student’s *T*-test with unequal variances on Microsoft Excel. Similar results were obtained for male and female pituitaries.

For nuclear density measurements, pituitary sections were stained with anti-Tpit antibodies (homemade rabbit polyclonal 1250B, dilution 1/100) or with Hoechst (SIGMA B2883) in the case of *Tpit*-/- pituitaries. After image acquisition IL nuclear density was analyzed using the Imaris Software and custom written MATLAB programs designed as follows: (1) the region was first outlined by hand to keep only the pituitary, (2) the position of each cell was identified via the fluorescent channel using the spot function from Imaris with background subtraction and fixed quality factor, (3) the area was calculated from the object thus created, and (4) cell density was counted as the number of spots over the object surface. The full processing *XTension* method is found at http://open.bitplane.com/Default.aspx?tabid=57&userid=337 with an example. Statistical significance was assessed using bilateral Student’s *T*-test with unequal variances on Microsoft Excel.

Total IL or AtT-20 protein extracts were used for Western blots^53^. Blots were first incubated with anti-mTOR (Cell Signaling Ab #2972, rabbit polyclonal, dilution 1/1000), anti-phosphoSer2481-mTOR (Cell Signaling Ab #2974, rabbit polyclonal, dilution 1/1000), or anti-Creb3l2 (NOVUS Biologicals NBP1-88697) antibodies, followed by peroxidase-coupled goat anti-rabbit (SIGMA, A6154, dilution 1/25,000).

### Cell culture

AtT-20, αT3, or GH3 cells were cultured in Dulbecco’s modified Eagle’s medium supplemented with 10% fetal bovine serum and antibiotics (penicillin/streptomycin). INS-1 cells were cultured in 1x RPMI 1640 medium (Wisent 350000CL) supplemented with 10 mM HEPES, pH 7.4, 1 mM Na pyruvate, 50 µM β-mercaptoethanol, 10% fetal bovine serum, and antibiotics (penicillin/streptomycin).

To generate stable transgenic cell populations, retroviruses were packed using the EcoPack 2-293 cells (Clontech, Mountain View, CA) and infections were performed as described^54^. Selection of retrovirus-infected cell populations was achieved with either 400 µg/ml Geneticin (Gibco, 11811-031) or 250 µg/ml Hygromycin B (Invitrogen, 10687-010). Resistant colonies were pooled to generate retrovirus-infected populations of about 1000 independent colonies. For double infections, cells were first infected by pLNCX2/3xFLAG-Creb3l2 and the resulting Creb3l2/Neo cells were re-infected by pLHCX/XBP1s and selected on media containing Geneticin/Hygromycin.

For genomic DNA extraction and quantification, AtT-20 cells were seeded in triplicates in 12-well plates (3 × 10^5^ cells/well) at day 0. At day 2, media were changed and at day 3, genomic DNA was extracted as for IL samples, resuspended in 50 µl of 10 mM Tris, pH 8.0 and quantitated using a fluorometer.

Total RNA was extracted using RNeasy Mini kit (Qiagen, 74104) following manufacturer’s recommendations. Experiments were done in triplicates. Statistical significance was assessed using bilateral Student’s *T*-test with unequal variances on Microsoft Excel.

For measurements of protein content, cells were plated as for DNA quantification. At day 3, cells were washed twice with 1xPBS, lysed with the EBC buffer (50 mM Tris–HCl pH 8.0, 170 mM NaCl, 0.5% NP-40, 50 mM NaF, 10% glycerol) and soluble protein concentration was measured by absorbance using the Bradford assay (Bio-Rad protein assay 500-0006). For each cell line, four wells were used for protein extractions and three for genomic DNA quantitation (as described above), used as indicator of cell number. The experiment was performed in quadruplicates 5–8 times. Statistical significance was assessed using bilateral Student’s *T*-test with unequal variances on Microsoft Excel.

ACTH release was measured using a commercially available ELISA kit (mdbioproducts M046006). AtT-20 cells grown on 12-well plates (3 × 10^5^ cells/well) for 48 h were placed on lipid-free low-serum medium (0.5% Dextran-coated charcoal treated FBS) for 12 h. Cells were then washed with serum-free medium and placed in 1 ml of serum-free medium containing CRH (10^−7^ M) or vehicle (1xPBS) for 7 h. The accumulated amount of ACTH released in the medium was measured following instructions of the manufacturer using 0.4 µl of medium (100 µl of a 1/250 dilution) per assay. Genomic DNA (quantified as above) was used as indicator of cell number. The experiment was done in duplicates at least three times. Statistical significance was assessed using bilateral Student’s *T*-test with unequal variances on Microsoft Excel.

Metabolic studies in different AtT-20 cell populations (respiration, lactate, and glucose levels) were performed as described^55^.

For transient transfections, αT3 (Fig. 2i), GH3 (Fig. 4f and Supplementary Fig. 4c, d), INS-1 (Fig. 3b and Supplementary Fig. 2b, c), or AtT-20 cells (Supplementary Fig. 2a) cells were plated in 12-well plates (3 × 10^5^ cells/well) the day preceding transfection and transfected with 1.4 µg of DNA, containing or not 100 ng of luciferase reporter construct with different amounts of expression vectors completed with empty expression vector (JA1394) using Lipofectamine reagent (Invitrogen, Carlsbad, CA, USA). For proteasome inhibition, 1.5 µM MG132 was added 5 h before cell harvesting.

### DNA vectors

Supplementary Data 3 lists reagents used to construct all expression vectors, including relevant PCR primers and used restriction sites.

The following retroviral vectors were used for generation of stable cell populations: pLNCX2 (JA1784), pLNCX2/XBP1s (JA2335), pLHCX (Clontech), pLHCX/XBP1s (JA2340), and pLNCX2/3xFLAG-Creb3l2 (JA2349).

For transient transfections, XBP1s and Creb3l2 (full-length or cleaved fragment) expression vectors (JA2343, JA2614, and JA2426, respectively) were constructed in the expression vector JA1394^18^. The coding sequences corresponding to XBP1s (*spliced* XBP1) and Creb3l2 (*full-length or cleaved* Creb3l2) were amplified from IL or AtT-20 cDNA by PCR. To obtain the 3xCreb3l2-RE-luciferase reporter (JA2422), we hybridized oligonucleotides 59-5/59-6 and then cloned their double-stranded DNA into the BamHI site located upstream of a minimal POMC promoter in plasmid JA290^56^.

To produce DN expression vectors (for transgenesis in vivo), the amphipathic acidic extension (AZIP) from the CMV A-CREB construct (Addgene catalog #33371)^57^ was PCR amplified with primers 30-585/25-208, introducing a NotI-restriction site on 5′ end and an XhoI site at the 3′ end. Second, Creb3l2 and XBP1 coding sequences starting at the leucine zipper domain were PCR amplified with primers 31-245/36-147 and 32-287/33-263 introducing a XhoI restriction site on 5′ end and an NotI site at the 3′ end. The, NotI-AZIP-XhoI DNA fragment was ligated to XhoI-Creb3l2-NotI or XhoI-XBP1-NotI and the resulting NotI-ACreb3l2-NotI or NotI-AXBP1-NotI fragments were subcloned in the vector JA1394/NotI, giving rise to plasmids JA2428 and JA2429, respectively. Finally, NotI-ACreb3l2-NotI or NotI-AXBP1-NotI DNA fragments from JA2428 and JA2429 were blunt-ended with DNA polymerase I large fragment (Klenow) and cloned into JA1508/EcoRI-Klenow downstream of the rat *Pomc* promoter and upstream of the SV40 intron. The resulting plasmids JA2452 and JA2453 were digested by SalI to generate SalI-POMC_promoter-ACreb3l2-SV40-SalI (≈1.8 kb) and SalI-POMC_promoter-AXBP1-SV40-SalI (≈2.5 kb) DNA fragments used for oocyte microinjection.

### FACS analyses

IL cells were prepared and analyzed^52^ using the FACS Calibur cell sorter (BD BioSciences). FSC and SSC gates were set to exclude cell debris and doublets (Supplementary Fig. 5). Propidium iodide labeling (1 µg/10^6^ cells) was used to exclude dead cells. Data were analyzed with the Summit 4.3 software. Cell diameter of IL cells was inferred by extrapolating forward scatter fluorescence values to the standard curve made using a set of calibrated beads (Spherotech PPS-6K). Cell granularity was assessed based on the side scatter (SSC) measured by FACS. Size and granularity of AtT-20 and INS-1 cells were analyzed the same way. The experiments were repeated at least three times. Statistical significance was assessed using bilateral Student’s *T*-test with unequal variances on Microsoft Excel.

### RNA extraction and transcriptomic analyses

IL or AtT-20 cell RNA was extracted using RNeasy mini extraction columns (Qiagen) according to the manufacturer’s instructions. 100–200 ng (IL) of total RNA were utilized to generate cDNA using the Superscript III reverse transcriptase (Invitrogen, 18080-044) following the manufacturer’s recommendations. Resulting cDNAs were analyzed by qPCR using a SYBR Green master mix (ThermoFisher Scientific A25741) supplemented with 300 nM of each gene-specific primer pair (see Supplementary Table 1 for sequences). Transcriptomic (RNAseq) studies were performed in triplicates for different AtT-20 cell populations or in duplicates for mice, using pools of five 3 months old male IL tissues. For *WT* or *Tpit-KO* ILs, cDNA probes were hybridized on Affymetrix mouse Gene 1.0 ST arrays. Hybridization and scanning were done at the McGill University and Genome Québec Innovation Centre. RNASeq were done at the IRCM Molecular Biology facility following the TruSeq-stranded mRNA sample preparation guide (Illumina).

### Chromatin immunoprecipitation

ChIP experiments were performed in AtT-20 cells as previously described^58^ using 5 µg of antibody (anti-FLAG-M2, mouse monoclonal, Sigma F3165; anti-mouse XBP1, rabbit polyclonal M-186, Santa Cruz Sc-7160, anti-human Creb3l2, rabbit polyclonal, Novus Biologicals, NBP1-88697) per reaction. Equal amounts of purified rabbit or mouse IgG (rabbit IgG, Sigma G2018; mouse IgG, Sigma I5381) were used in control ChIP reactions. qPCR analyses were done using SYBR Green master mix (ThermoFisher Scientific A 25741) supplemented with 300 nM of primer pairs (see Supplementary Table 1 for primer sequences). Pools of DNA from several independent ChIP experiments were utilized to make libraries for ChIPseq. The libraries and flowcells were prepared at the IRCM Molecular Biology Core facility following Illumina recommendations (Illumina, San Diego, CA) and then sequenced on Hiseq 2000 at the Génome Québec Innovation Centre. The generation of IgG, Flag, Tpit, H3K4me1, H3K27ac ChIPseq profiles in AtT-20 cells was already described^24^. ATACseq data in AtT-20 cells and pituitary corticotropes and melanotropes were described^59^ and are available on GEO as GSE87185.

### Bioinformatics processing and analysis of genomic data

The FlexArray software developed at McGill University (http://genomequebec.mcgill.ca/FlexArray) was used to analyze transcriptomic data (GC-RMA normalization and Scatter plots). Differentially expressed genes were extracted by fixing a *P*-value threshold (*P* ≤ 0.001) using the Local-pooled-error (LPE) test.

Finding of enriched functional-related gene groups was done using Gene Ontology tool from the Database for Annotation, Visualization, and Integrated Discovery (DAVID)^60^ or AmiGO1 (http://amigo1.geneontology.org/cgi-bin/amigo/term_enrichment)^61,62^. *P*-values on DAVID (Fig. 5a) correspond to EASE Score, a modified (more conservative) Fisher Exact *P*-value, while *P*-values on AmiGO (Figs. 2b, 4e and Supplementary Fig. 3g) were calculated with GO::TermFinder using the hypergeometric distribution.

ChIPseq peak finding and sequence analysis were described previously^24^. For de novo motif analysis, 120 bp of sequences surrounding the peaks were extracted from the University of California at Santa Cruz Web site^63^ and processed using HOMER^64^. It uses ZOOPS scoring (zero or one occurrence per sequence) coupled with the hypergeometric enrichment calculations (or binomial) to determine motif enrichment. Graphical representations of the position weight matrices obtained from these analyses were generated with WebLogo (http://demo.tinyray.com/weblogo)^65^. Peaks were assigned to the closest gene with the AnnotatePeaks.pl Homer command. To visualize ChIPseq profiles, we used the Integrative Genome Viewer tool^66^.

RNAseq reads were trimmed with Trimmomatic v0.22^67^ and aligned against the mm10 mouse genome using Tophat (v2.0.8). Raw read counts per gene were calculated with HTSeq-count from HTSeq (v0.5.4) (http://www-huber.embl.de/users/anders/HTSeq/). The raw counts were normalized relative to the library size, and tested for differential expression using the R package DESeq^68^. Genes were considered to be expressed if the average normalized count across the samples was above 20.

For unsupervised clustering, a table containing gene expression fold changes in different LOF or GOF conditions was uploaded into Cluster 3.0 software (enhanced version of Cluster, originally described in ref. ^69^). After normalization, we applied *K*-means for clustering using Euclidean distance similarity metrics. We determined that four clusters is the most segregating setting for our dataset. The gene lists extracted from those four clusters were uploaded into the DAVID website^60^ to search for enriched biological processes.

### Reporting summary

Further information on research design is available in the Nature Research Reporting Summary linked to this article.

## Supplementary information

Supplementary Information

Supplementary Data 1/2/3

Reporting Summary

## Data Availability

All genomic data have been deposited on Gene Expression Omnibus (GEO) under accession number GSE132324.
