# Supplementary material for: Pituitary cell translation and secretory capacities are enhanced cell autonomously by the transcription factor Creb3l2
Source: Nat Commun. 2019 Sep 3;10:3960. doi: 10.1038/s41467-019-11894-3 (PMC6722061; doi:10.1038/s41467-019-11894-3)
Supplement: Supplementary file 1 — Supplementary Information [file 41467_2019_11894_MOESM1_ESM.pdf]

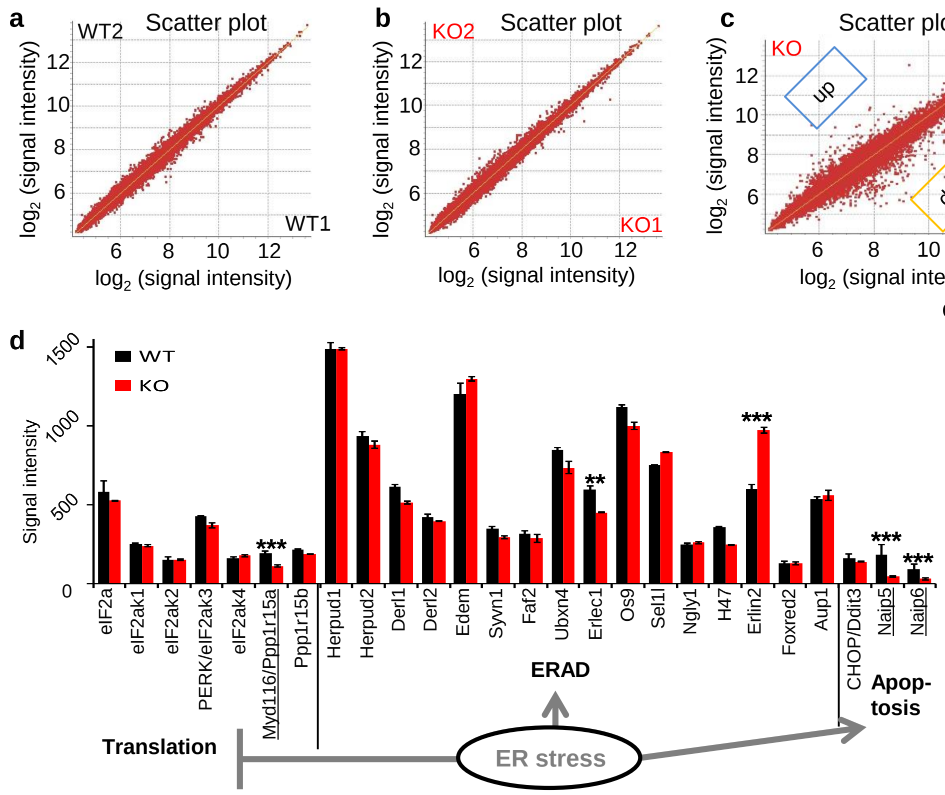

e

Rank	Gene Symbol	Mean WT	Mean KO	Fold change	*P*-value
	Tpit	1709	99	0.06	0.0E+00
1	*Nor1*	358	75	0.21	0.0E+00
2	*Nurr1*	1130	239	0.21	0.0E+00
3	*Fos*	369	86	0.23	0.0E+00
4	*Fosb*	194	50	0.26	0.0E+00
5	*Egr1*	293	76	0.26	0.0E+00
6	*Nur77*	2190	573	0.26	0.0E+00
7	***Creb3l2***	1229	456	0.37	0.0E+00
8	*Mash1*	1679	641	0.38	0.0E+00
9	*Pax7*	751	291	0.39	0.0E+00

f **Tissue distribution of the OASIS family membres, Tpit and POMC (from Thorrez et al.[1])**

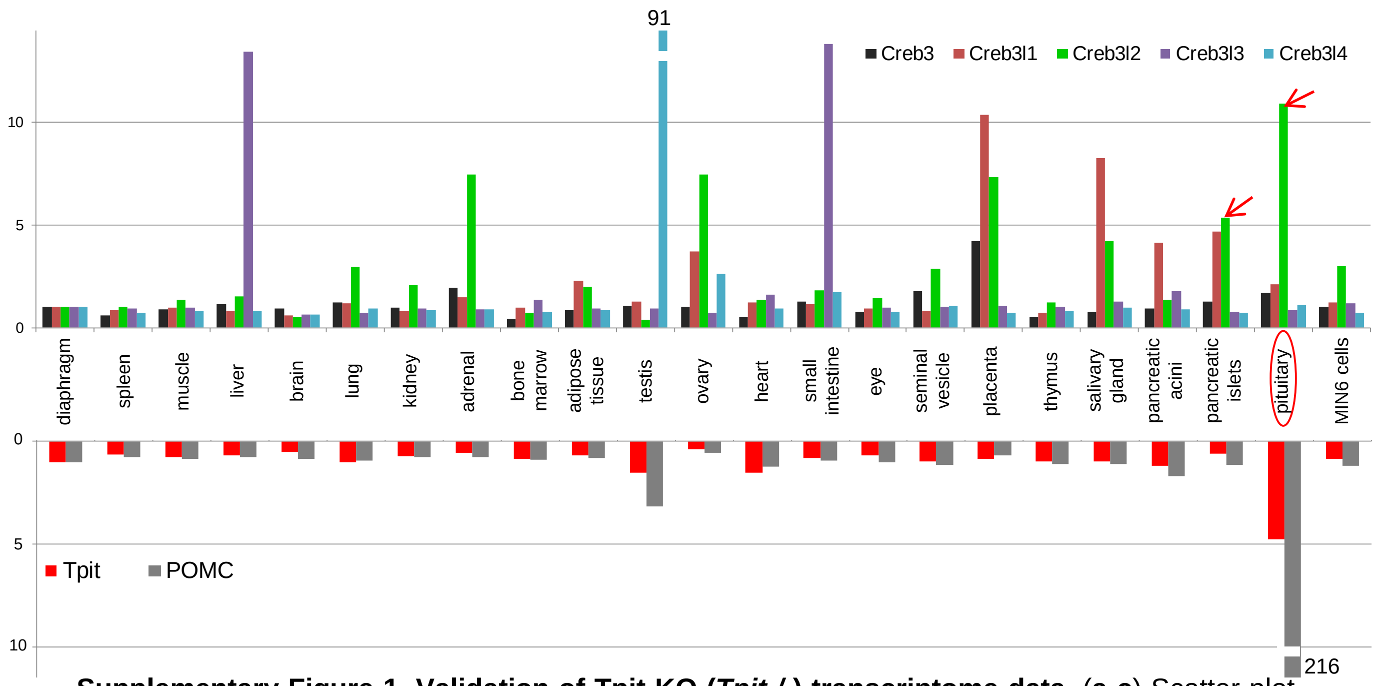

Supplementary Figure 1. Validation of Tpit KO (*Tpit-/-*) transcriptome data. (**a-c**) Scatter plot comparisons of 2 WT (**a**) and 2 KO (**b**) pituitary intermediate lobe (IL) transcriptomes showing the reproducibility of microarray experiments and comparison of WT *vs* Tpit KO pituitary IL transcriptomes (**c**). (**d**) Expression (Affymetrix microarray signals) of translation regulators, ERAD and apoptotic genes in WT (black bars) and KO (red bars) ILs. *P*-values determined using local-pooled-error (LPE) test. Compared to controls (WT): ** *P< 0.005*, *** *P< 0.0005*. (**e**) List of 10 most downregulated transcription factor genes in *Tpit*-deficient ILs. (**f**) Expression of the OASIS family members, *Tpit* and *POMC* in different tissues and cell lines.

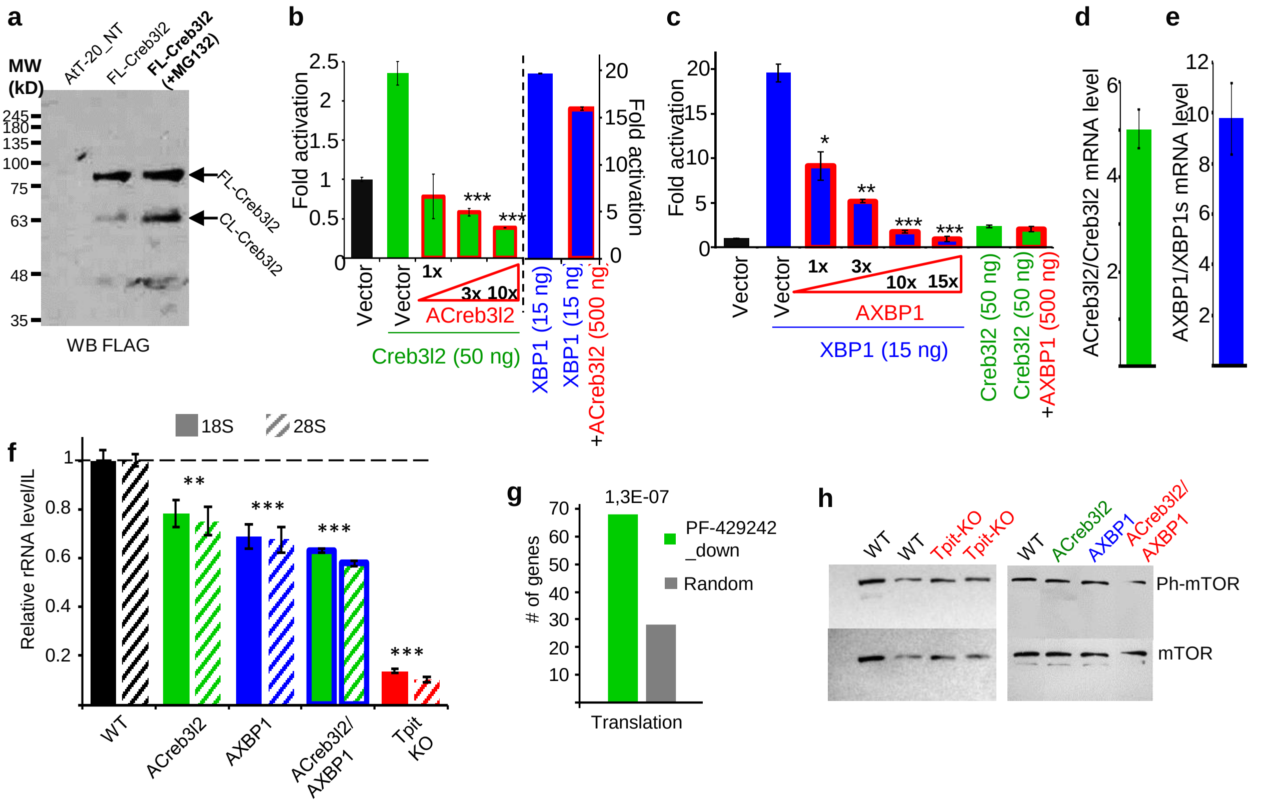

Supplementary Figure 2. Efficient and specific inhibition of Creb3l2 and XBP1 activity using acidic dominant-negative constructs (AZIP). (**a**) Expression (Flag Western blot) of FL-Creb3l2 in AtT-20 cells results in production of both FL and cleaved (CL) active forms of Creb3l2. Treatment with the proteasome inhibitor MG132 leads to stabilization of Creb3l2. (**b**) Inhibition of Creb3l2, but not XBP1, activity by overexpression of ACreb3l2. (**c**) Inhibition of XBP1, but not Creb3l2, activity by overexpression of AXBP1. The 3xCreb3l2/XBP1-RE *Luciferase* reporter was used to assess Creb3l2 or XBP1 activity upon transfection into INS-1 cells. (**d-e**) RT-qPCR quantification of ACreb3l2 and AXBP1 transcripts relative to the endogenous Creb3l2 and XBP1s transcripts in ILs of the transgenic mice reported in Fig. 3. (**f**) RT-qPCR assessment of rRNA 18S and 28S contents in WT, LOF transgenics and *Tpit* KO ILs. Data represent the average ±SEM of 4 pools of 5-10 ILs. Statistical significance was assessed using bilateral Student's *T*-test with unequal variances. ** *P<0.005*, *** *P<0.0005*. (**g**) Enrichment of translation genes in transcriptomes of plasmablasts treated for 72h with the selective S1P (S1 protease responsible for Creb3l2 cleavage) inhibitor PF-429242 (analysis of raw data from Al-Maskari et al.[2]). (**h**) mTORC1 status is not affected by Tpit KO or Creb3l2/XBP1 downregulation (ACreb3l2, AXBP1). Western blot showing similar active mTOR (phospho Ser-2481) to total mTOR levels in IL protein extracts from indicated mutant mice.

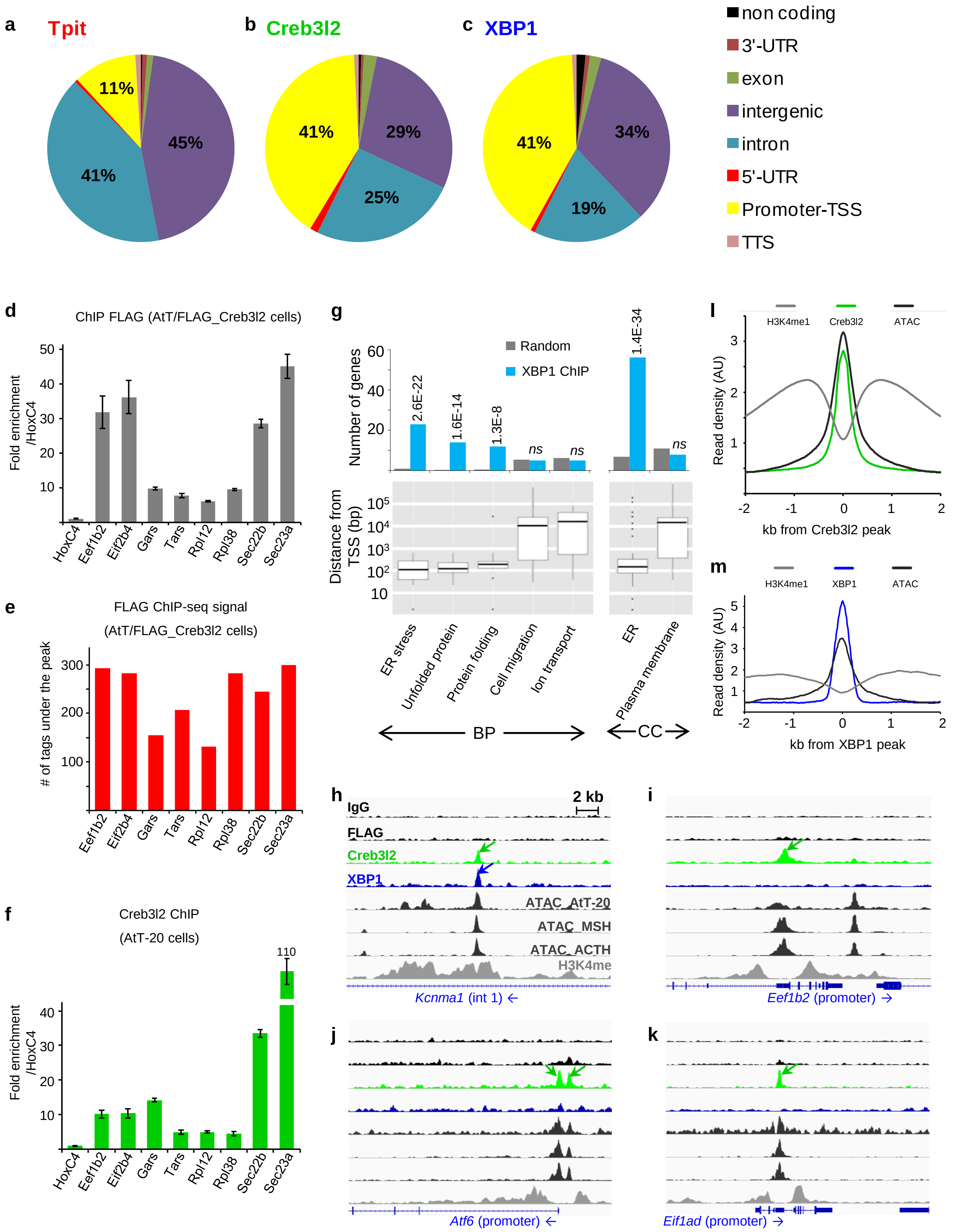

a Tpit
b Creb3l2
c XBP1
11%
45%
41%
41%
29%
25%
41%
34%
19%
non coding
3'-UTR
exon
intergenic
intron
5'-UTR
Promoter-TSS
TTS
d ChIP FLAG (AtT/FLAG_Creb3l2 cells)
Fold enrichment /HoxC4
HoxC4
Eef1b2
Eif2b4
Gars
Tars
Rpl12
Rpl38
Sec22b
Sec23a
e FLAG ChIP-seq signal
(AtT/FLAG_Creb3l2 cells)
# of tags under the peak
f Creb3l2 ChIP
(AtT-20 cells)
110
g
Number of genes
Random
XBP1 ChIP
2.6E-22
1.6E-14
1.3E-8
ns
1.4E-34
Distance from TSS (bp)
ER stress
Unfolded protein
Protein folding
Cell migration
Ion transport
ER
Plasma membrane
BP
CC
l
H3K4me1
Creb3l2
ATAC
Read density (AU)
kb from Creb3l2 peak
m
XBP1
kb from XBP1 peak
h
IgG
2 kb
FLAG
ATAC_AtT-20
ATAC_MSH
ATAC_ACTH
H3K4me
Kcnma1 (int 1) ←
i
Eef1b2 (promoter) →
j
Atf6 (promoter) ←
k
Eif1ad (promoter) →

Supplementary Figure 3. Creb3l2 and XBP1 target the promoters of translation and ER biogenesis genes, respectively. (**a-c**) Genomic distribution of Tpit, Creb3l2 and XBP1 ChIPseq peaks. (**d-f**) ChIP-qPCR validation of Creb3l2 ChIPseq. Data for 8 representative promoter loci are shown as assessed by FLAG antibody ChIP-qPCR (**d**), FLAG antibody ChIPseq (**e**) and Creb3l2 antibody ChIP-qPCR (**f**). (**g**) GO terms of genes associated with TSS proximal (≤1 kb) XBP1 peaks. Peaks were assigned to the closest gene with the AnnotatePeaks Homer command. Bars represent the number of genes in each category associated with XBP1 peaks (blue) or random occurrence of genes in each category (grey). *P*-values relative to random occurrence determined by using hypergeometric distribution provided by AmiGO are shown above the bars; ns, not significant. The bottom panels provide boxplot representation of the distance to TSS for XBP1 peaks of each GO category revealing promoter-proximal associations for groups with significant associations. Center lines show medians; box limits indicate the twenty-fifth and seventy-fifth percentiles; whiskers extend to 1.5 times the interquartile range from the twenty-fifth to seventy-fifth percentiles. BP: biological process, CC: cellular component. (**h-k**) ChIPseq profiles at regulatory sequences of genes targeted by Creb3l2 and/or XBP1. ChIPseq patterns are shown for control IgG, Flag, Creb3l2, XBP1, H3K4me1 and ATACseq. (**l,m**) Average profiles of H3K4me1 ChIPseq and ATACseq at Creb3l2 (**l**) or XBP1 (**m**) peaks.

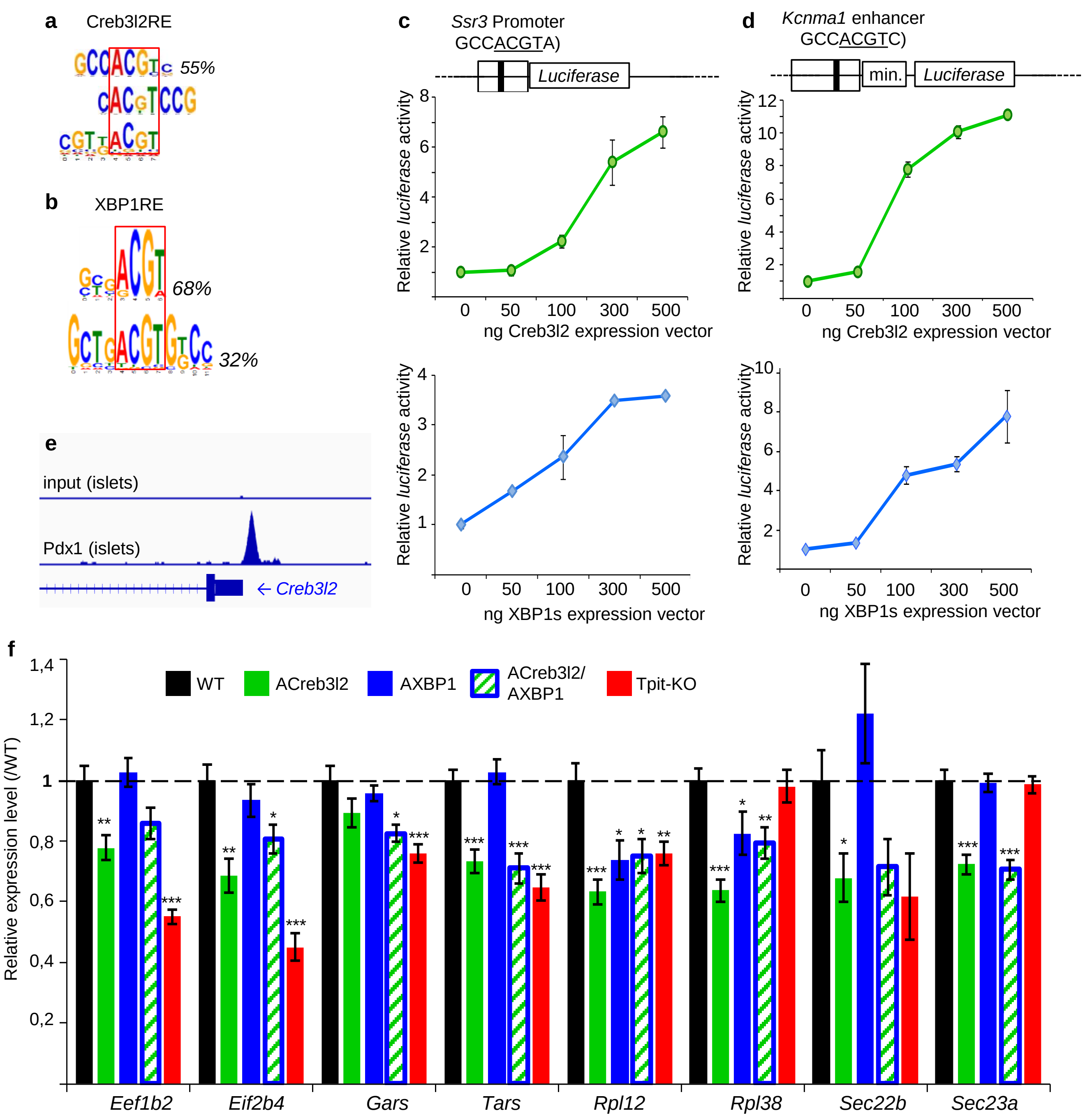

Supplementary Figure 4. Creb3l2 and XBP1 activate transcription through binding of a similar sequence motif. (**a-b**) Creb3l2-RE and XBP1-RE consensus identified by *de novo* motif searches in sequences bound by Creb3l2 and XBP1. (**c-d**) The transcriptional activity of Creb3l2 and XBP1 assessed by transfection in GH3 cells using two pGL4.10-*luciferase* reporters. Schematic representation of *Luciferase* reporters containing the *Ssr3* promoter (**c**) or *Kcnma1* enhancer (**d**) and dose-response curves. (**e**) ChIPseq profiles of Pdx1 at regulatory sequences of Creb3l2 in pancreatic beta cells. Data from http://chip-atlas.org (the Pdx cistrome of pancreatic islets, ERX103428, ERX103429). (**f**) RT-qPCR validation of RNAseq analyses at 8 representative loci. Statistical significance was assessed using bilateral Student's *T*-test with unequal variances. * *P<0.05, ** P<0.005, *** P<0.0005* compared to WT.

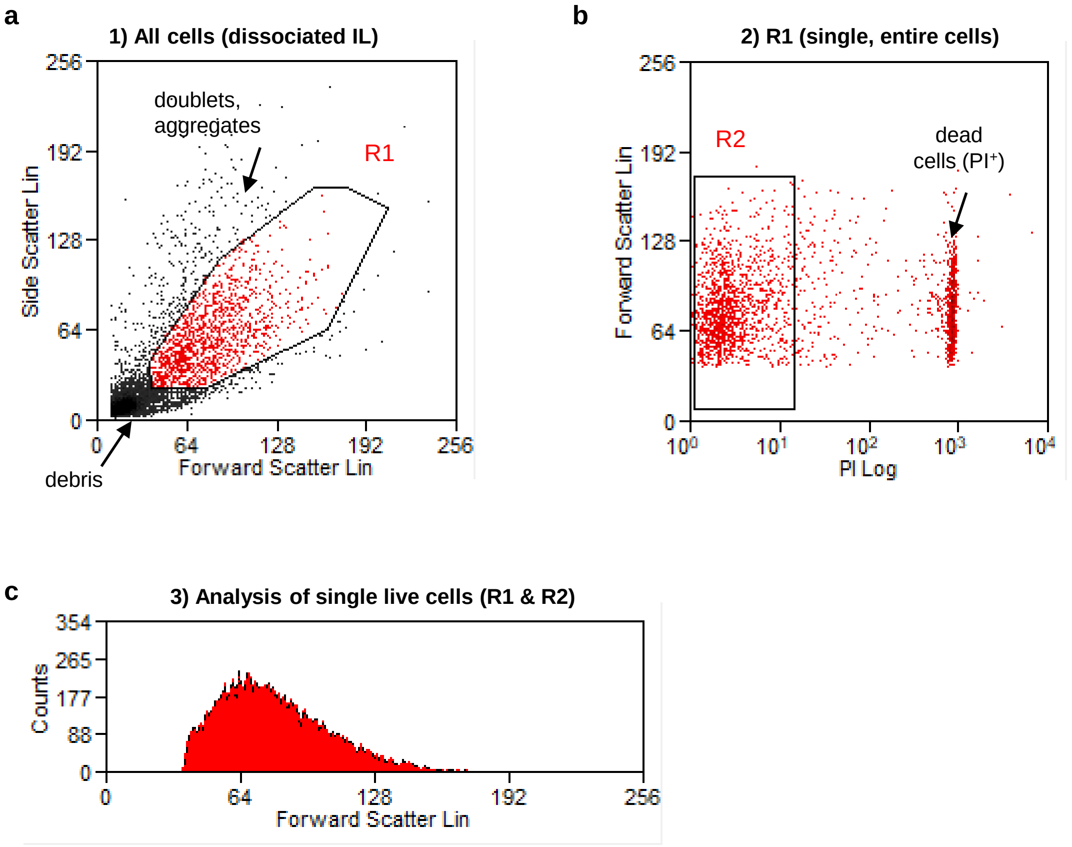

Supplemenrtary Figure 5. Gating strategy used in FACS experiments. Followed IL dissociation cells are stained with propidium iodide (PI) and analyzed by FACS Calibur cell sorter (BD BioSciences). Gating and data analysis are done using the Summit 4.3 software. (**a**) A first gate (R1) is drawn to exclude cell debris and doublets or cell aggregates. (**b**) PI-positive (dead) cells are then excluded from the R1 gate, giving the R2 population. (**c**) Cells from R1 & R2 gates (single, live cells) are analyzed for different properties (FSC, reflecting cell size in the given example).

Supplementary Table 1.

Primers used for RT-qPCR experiments

Species	Gene	Orientation	Sequence	Product size (bp)	Name
Mus musculus	*18S*	sens	CTCAACACGGGAAACCTCAC	110	20-2465
		antisens	CGCTCCACCAACTAAGAACG		20-2466
	28S	sens	CTAAATACCGGCACGAGACC	88	20-2467
		antisens	TTCACGCCCTCTTGAACTCT		20-2468
	ATF4	sens	ATGAGCTTCCTGAACAGCGA	223	20-1816
		antisens	AAGGCATCCTCCTTGCCGGT		20-1817
	ATF6	sens	TGGGAGTGAGCTGCAAGTGTAT	140	22-1260
		antisens	TGTTGTGGGTGGTAGCTGGTAA		22-1261
	Creb3	sens	AACAAGGTGCAGCGTTTGGA	127	20-2120
		antisens	ACACGAGGACCAGAACACAAGT		22-1282
	Creb3l2 (bZIP)	sens	TGAGGAGAAGGCCCTGAAGAAA	150	22-1294
		antisens	ACCTTCTTCCGAAGCTCCAAGT		22-1295
	Creb3l2 (bZIP & AZIP)	sens	AGGTGCTGGAGAACACCAAT	107	20-2095
		antisens	AAGTCTGTGTGCCAGCTAAC		20-2336
	Dnajb9	sens	AGCCCTGATGCTGAAGCAAA	130	20-1818
		antisens	TGCCTCTTTGTCCTTTGCCA		20-1819
	Eef1b2	sens	CGGGAGTGAAGAAATCTTTG	132	20-2220
		antisens	CCTCACTTTCCTCCTCATC		19-341
	Eif2b4	sens	GAAGGAAGGCACATGCTC	121	18-501
		antisens	GTGCATGAGCTCCCAATAG		19-342
	Gars	sens	CAAAGCTAGTGCTGGAGTATC	152	21-597
		antisens	CTGGAATCTCTTCACACTGAC		21-598
	Rpl12	sens	CAGAAGAACATTAAACACAGTG	126	22-601
		antisens	TGCAGTACCCAGGATCTC		18-696
	Rpl38	sens	GAGGAGATCAAGGACTTTCTG	156	21-599
		antisens	CTTCAGCTTCTCTGCCTTT		19-473
	Sec22b	sens	ATGTGCAGAGGATCATGG	120	18-577
		antisens	CATCCTGGCGGTATTTCT		18-578
	Sec23a	sens	TCCTCATGGACACGTTCTTCCA	116	22-1280
		antisens	TTGCAGAAGGTGCCGGAAAT		20-2117
	TAF8	sens	ACGCTCTTCATATCAGCACGGA	109	22-1286
		antisens	ATGACGCTCTCCTCGCCATT		20-2121
	Tars	sens	GATTGCCATCCTCACAGAAA	151	20-2459
		antisens	AGTGTCCGCCATGAATTTAG		20-2460
	TBP	sens	ACAGGACTTACTCCACAGCCTA	200	22-338
		antisens	AGTTGCTACTGCCTGCTGTT		20-838
	Tpit	sens	TGAAATGATCGTGACCAAGAACGG	145	24-257
		antisens	TTCACCATTGACGTACTTCCAGCG		24-258
	XBP1s (bZIP)	sens	GAAAGCCCGGATGAGCGA	236	18-613
		antisens	CACCTGCTGCGGACTC		16-68
	XBP1s (bZIP & AZIP)	sens	GAGTCCGCAGCAGGTG	83	16-66
		antisens	GAATCTGAAGAGGCAACAGTG		21-453
Xenopus laevis	*ATF4*	sens	ACGCAAGGATTTCTTCACTCCG	146	22-1222
		antisens	ATGGCTGAGCCTGGTGTT		18-423
	β-actin	sens	GCCATCTTTCCTTGGTATG	117	19-288
		antisens	CCACCAGACAGAACAGTA		18-426
	Creb3l2	sens	ACAGCCTGGAGAAGAGGGTT	112	20-2097
		antisens	AGACGCTGGAGTTGCTGAAGTA		22-1240
	POMC	sens	CCTCAGCAGTGAAGATGGTGTT	96	22-1223
		antisens	TGCAAATGTCCGTTTCCTGG		20-2077
	Sec23a	sens	CACTTTGCCAGACAAGAC	149	18-427
		antisens	TGGAAGAAGGTGTCCATAA		19-289
	Sec23b	sens	ATGCACCTATTCCTCAAGGTGG	170	22-1221
		antisens	TACTGCCGCTGCTTCTTGAT		20-2070
	XBP1s	sens	GAGTCCGCAGCAGGAG	83	16-67
		antisens	GAGTCTGAAGTGTCAGGGCT		20-2066

Primers used for ChIP-qPCR experiments (promoters)

Gene	Orientation	Sequence	Product size (bp)	Name
Eef1b2	sens	GGCGTTTCCCTTCGTTTC	131	18-679
	antisens	ACCACTGCGACTTGGTAA		18-680
Eif2b4	sens	CCATGACTGTGGGTCCTA	181	18-681
	antisens	GGGAACCCAACATCCTTT		18-682
Gars	sens	GCGTCCTCTTCACACATTT	149	19-461
	antisens	CCCGGTGCATGATGAAATA		19-462
HoxC4	sens	CCTACACAGACTGCAACAC	160	19-468
	antisens	CAGCTCGACTCCAATGTTT		19-469
Rpl12	sens	CAACGGTGCAACTTTCTTC	100	19-464
	antisens	CGACTTTGACCTCGTTGG		18-685
Rpl38	sens	GCCTAACGCCATTGCTATAA	132	20-2453
	antisens	GCCCAACAGAGGAAACTG		18-686
Sec22b	sens	CTTGCAGAGACACCACTTAG	149	20-2454
	antisens	GACTTCAGAGCTTTCCACTAC		21-596
Sec23a	sens	CTCCAAAGCCCTGTAGCA	173	18-687
	antisens	CCTCTTCCGGGACTGTTC		18-688
Tars	sens	GCCAATAGAATAGCCGAGAAT	148	21-595
	antisens	GTCCAAGCAGCTCAAGTT		18-683

References to supplementary figures

1. Thorrez, M. et al. Tissue-specific disallowance of housekeeping genes: the other face of cell differentiation. Genome Res 21, 95-105, doi:10.1101/gr.109173.110 (2010).

2. Al-Maskari, M. *et al.* Site-1 protease function is essential for the generation of antibody secreting cells and reprogramming for secretory activity. *Scientific reports* **8**, 14338, doi:10.1038/s41598-018-32705-7 (2018).
