# Supplementary material for: Pituitary cell translation and secretory capacities are enhanced cell autonomously by the transcription factor Creb3l2
Source: Nat Commun. 2019 Sep 3;10:3960. doi: 10.1038/s41467-019-11894-3 (PMC6722061; doi:10.1038/s41467-019-11894-3)
Supplement: Supplementary file 3 — Reporting Summary [file 41467_2019_11894_MOESM3_ESM.pdf]

nature research

Corresponding author(s): Jacques Drouin and Konstantin Khetchoumian

Last updated by author(s): Jul 23, 2019

Reporting Summary

Nature Research wishes to improve the reproducibility of the work that we publish. This form provides structure for consistency and transparency in reporting. For further information on Nature Research policies, see Authors & Referees and the Editorial Policy Checklist.

Statistics

For all statistical analyses, confirm that the following items are present in the figure legend, table legend, main text, or Methods section.

n/a	Confirmed	
☐	☒	The exact sample size (n) for each experimental group/condition, given as a discrete number and unit of measurement
☐	☒	A statement on whether measurements were taken from distinct samples or whether the same sample was measured repeatedly
☐	☒	The statistical test(s) used AND whether they are one- or two-sided<br>*Only common tests should be described solely by name; describe more complex techniques in the Methods section.*
☒	☐	A description of all covariates tested
☒	☐	A description of any assumptions or corrections, such as tests of normality and adjustment for multiple comparisons
☒	☐	A full description of the statistical parameters including central tendency (e.g. means) or other basic estimates (e.g. regression coefficient) AND variation (e.g. standard deviation) or associated estimates of uncertainty (e.g. confidence intervals)
☒	☐	For null hypothesis testing, the test statistic (e.g. F, t, r) with confidence intervals, effect sizes, degrees of freedom and P value noted<br>*Give P values as exact values whenever suitable.*
☒	☐	For Bayesian analysis, information on the choice of priors and Markov chain Monte Carlo settings
☒	☐	For hierarchical and complex designs, identification of the appropriate level for tests and full reporting of outcomes
☒	☐	Estimates of effect sizes (e.g. Cohen's d, Pearson's r), indicating how they were calculated

Our web collection on statistics for biologists contains articles on many of the points above.

Software and code

Policy information about availability of computer code

Data collection	Sequencing data were collected by Illumina HiSeq2000 or 2500 softwares.
Data analysis	FlexArray (microarrays), HOMER, Samtools, STAR 2.5, Trimmomatic 0.36, picard 2.17.3, MACS, Bowtie v.2.3.1, Deseq, Deseq2 (in Bioconductor), htseq-count, cufflinks 2.2.4, R_Bioconductor v3.5.1_3.7, Cluster v3.0, TreeView, Summit v4.3 (FACS).

For manuscripts utilizing custom algorithms or software that are central to the research but not yet described in published literature, software must be made available to editors/reviewers. We strongly encourage code deposition in a community repository (e.g. GitHub). See the Nature Research guidelines for submitting code & software for further information.

Data

Policy information about availability of data

All manuscripts must include a data availability statement. This statement should provide the following information, where applicable:

- Accession codes, unique identifiers, or web links for publicly available datasets
- A list of figures that have associated raw data
- A description of any restrictions on data availability

All the sequencing data generated in this work are deposited on GEO as GSE132324

Field-specific reporting

Please select the one below that is the best fit for your research. If you are not sure, read the appropriate sections before making your selection.

☒ Life sciences ☐ Behavioural & social sciences ☐ Ecological, evolutionary & environmental sciences

Life sciences study design

All studies must disclose on these points even when the disclosure is negative.

Sample size	No statistical methods were used to predetermine sample size
Data exclusions	There was no exclusion/inclusion of samples or animals in the analysis
Replication	RT-qPCR on tissues were done in duplicates on pools of 5-10 IL cDNA samples per genotype (mice) or per condition (Xenopus).<br>RT-qPCR on AtT-20 cells were done in duplicates on 3 independent cDNA samples per genotype.<br>Transfections with luciferase reporters were done in duplicates at least 3 times.<br>RNAseq on tissues or AtT-20 cells were done in duplicates or triplicates. For tissues, IL RNA pools from 5 animals per genotypes were used.<br>The measures of translation rates (SunSET) were done on pools of 4-6 ILs at least 5 times.<br>The measures of IL ER content were done on pools of 3-5 ILs at least 3 times.<br>The measures of IL RNA content were done on pools of 5-10 ILs at least 6 times.<br>AtT-20 cell total protein content measures were done in quadruplicates 5-8 times.<br>ACTH secretion tests on different AtT-20 cell lines were done in duplicates at least 3 times.<br>Total RNA content in different AtT-20 cell lines was measured in triplicates.<br>Each western blot was performed 3 times.<br>Creb3l2 immunohistofluorescence was done 2 times on pituitary sections from 2 WT and 2 Tpit-KO mice.
Randomization	There was no randomization of experiments
Blinding	Investigators were not blinded during experiments and analysis. Genotyping was done before performing the experiments.

Behavioural & social sciences study design

All studies must disclose on these points even when the disclosure is negative.

Study description	*Briefly describe the study type including whether data are quantitative, qualitative, or mixed-methods (e.g. qualitative cross-sectional, quantitative experimental, mixed-methods case study).*
Research sample	*State the research sample (e.g. Harvard university undergraduates, villagers in rural India) and provide relevant demographic information (e.g. age, sex) and indicate whether the sample is representative. Provide a rationale for the study sample chosen. For studies involving existing datasets, please describe the dataset and source.*
Sampling strategy	*Describe the sampling procedure (e.g. random, snowball, stratified, convenience). Describe the statistical methods that were used to predetermine sample size OR if no sample-size calculation was performed, describe how sample sizes were chosen and provide a rationale for why these sample sizes are sufficient. For qualitative data, please indicate whether data saturation was considered, and what criteria were used to decide that no further sampling was needed.*
Data collection	*Provide details about the data collection procedure, including the instruments or devices used to record the data (e.g. pen and paper, computer, eye tracker, video or audio equipment) whether anyone was present besides the participant(s) and the researcher, and whether the researcher was blind to experimental condition and/or the study hypothesis during data collection.*
Timing	*Indicate the start and stop dates of data collection. If there is a gap between collection periods, state the dates for each sample cohort.*
Data exclusions	*If no data were excluded from the analyses, state so OR if data were excluded, provide the exact number of exclusions and the rationale behind them, indicating whether exclusion criteria were pre-established.*
Non-participation	*State how many participants dropped out/declined participation and the reason(s) given OR provide response rate OR state that no participants dropped out/declined participation.*
Randomization	*If participants were not allocated into experimental groups, state so OR describe how participants were allocated to groups, and if allocation was not random, describe how covariates were controlled.*

Ecological, evolutionary & environmental sciences study design

All studies must disclose on these points even when the disclosure is negative.

Study description	*Briefly describe the study. For quantitative data include treatment factors and interactions, design structure (e.g. factorial, nested, hierarchical), nature and number of experimental units and replicates.*
Research sample	*Describe the research sample (e.g. a group of tagged Passer domesticus, all Stenocereus thurberi within Organ Pipe Cactus National Monument), and provide a rationale for the sample choice. When relevant, describe the organism taxa, source, sex, age range and*

any manipulations. State what population the sample is meant to represent when applicable. For studies involving existing datasets, describe the data and its source.

Sampling strategy

Note the sampling procedure. Describe the statistical methods that were used to predetermine sample size OR if no sample-size calculation was performed, describe how sample sizes were chosen and provide a rationale for why these sample sizes are sufficient.

Data collection

Describe the data collection procedure, including who recorded the data and how.

Timing and spatial scale

Indicate the start and stop dates of data collection, noting the frequency and periodicity of sampling and providing a rationale for these choices. If there is a gap between collection periods, state the dates for each sample cohort. Specify the spatial scale from which the data are taken

Data exclusions

If no data were excluded from the analyses, state so OR if data were excluded, describe the exclusions and the rationale behind them, indicating whether exclusion criteria were pre-established.

Reproducibility

Describe the measures taken to verify the reproducibility of experimental findings. For each experiment, note whether any attempts to repeat the experiment failed OR state that all attempts to repeat the experiment were successful.

Randomization

Describe how samples/organisms/participants were allocated into groups. If allocation was not random, describe how covariates were controlled. If this is not relevant to your study, explain why.

Blinding

Describe the extent of blinding used during data acquisition and analysis. If blinding was not possible, describe why OR explain why blinding was not relevant to your study.

Did the study involve field work? ☐ Yes ☐ No

Field work, collection and transport

Field conditions

Describe the study conditions for field work, providing relevant parameters (e.g. temperature, rainfall).

Location

State the location of the sampling or experiment, providing relevant parameters (e.g. latitude and longitude, elevation, water depth).

Access and import/export

Describe the efforts you have made to access habitats and to collect and import/export your samples in a responsible manner and in compliance with local, national and international laws, noting any permits that were obtained (give the name of the issuing authority, the date of issue, and any identifying information).

Disturbance

Describe any disturbance caused by the study and how it was minimized.

Reporting for specific materials, systems and methods

We require information from authors about some types of materials, experimental systems and methods used in many studies. Here, indicate whether each material, system or method listed is relevant to your study. If you are not sure if a list item applies to your research, read the appropriate section before selecting a response.

Materials & experimental systems

n/a	Involved in the study
☐	☒ Antibodies
☐	☒ Eukaryotic cell lines
☒	☐ Palaeontology
☐	☒ Animals and other organisms
☒	☐ Human research participants
☒	☐ Clinical data

Methods

n/a	Involved in the study
☐	☒ ChIP-seq
☐	☒ Flow cytometry
☒	☐ MRI-based neuroimaging

Antibodies

Antibodies used

Listed in Materials and Methods.

Validation

Describe the validation of each primary antibody for the species and application, noting any validation statements on the manufacturer's website, relevant citations, antibody profiles in online databases, or data provided in the manuscript.

Eukaryotic cell lines

Policy information about cell lines

Cell line source(s)

AtT-20 cells obtained from the late E. Herbert in 1981 and maintained in this lab since.

Authentication	Cells were not authenticated by karyotyping but are routinely assessed for POMC expression and responsiveness to CRH and glucocorticoids, the hallmarks biological activities of corticotrope cells that constitute the basis for using this model.
Mycoplasma contamination	No, tested on a yearly basis.
Commonly misidentified lines (See ICLAC register)	No commonly misidentified cell lines were used in this study.

Palaeontology

Specimen provenance	*Provide provenance information for specimens and describe permits that were obtained for the work (including the name of the issuing authority, the date of issue, and any identifying information).*
Specimen deposition	*Indicate where the specimens have been deposited to permit free access by other researchers.*
Dating methods	*If new dates are provided, describe how they were obtained (e.g. collection, storage, sample pretreatment and measurement), where they were obtained (i.e. lab name), the calibration program and the protocol for quality assurance OR state that no new dates are provided.*

☐ Tick this box to confirm that the raw and calibrated dates are available in the paper or in Supplementary Information.

Animals and other organisms

Policy information about studies involving animals; ARRIVE guidelines recommended for reporting animal research

Laboratory animals	Stated in Methods: mice on C57Bl/6 or Balb/c backgrounds; Xenopus laevis.
Wild animals	Not used in this study.
Field-collected samples	Not used in this study.
Ethics oversight	Animal studies were approved by the IRCM Animal Ethics Committee following Canadian regulations.

Note that full information on the approval of the study protocol must also be provided in the manuscript.

Human research participants

Policy information about studies involving human research participants

Population characteristics	*Describe the covariate-relevant population characteristics of the human research participants (e.g. age, gender, genotypic information, past and current diagnosis and treatment categories). If you filled out the behavioural & social sciences study design questions and have nothing to add here, write "See above."*
Recruitment	*Describe how participants were recruited. Outline any potential self-selection bias or other biases that may be present and how these are likely to impact results.*
Ethics oversight	*Identify the organization(s) that approved the study protocol.*

Note that full information on the approval of the study protocol must also be provided in the manuscript.

Clinical data

Policy information about clinical studies

All manuscripts should comply with the ICMJE guidelines for publication of clinical research and a completed CONSORT checklist must be included with all submissions.

Clinical trial registration	*Provide the trial registration number from ClinicalTrials.gov or an equivalent agency.*
Study protocol	*Note where the full trial protocol can be accessed OR if not available, explain why.*
Data collection	*Describe the settings and locales of data collection, noting the time periods of recruitment and data collection.*
Outcomes	*Describe how you pre-defined primary and secondary outcome measures and how you assessed these measures.*

ChIP-seq

Data deposition

☒ Confirm that both raw and final processed data have been deposited in a public database such as GEO.

☒ Confirm that you have deposited or provided access to graph files (e.g. BED files) for the called peaks.

Data access links
May remain private before publication.

GEO GSE132324

Files in database submission

GSE132324 Super-serie
GSE132320 Cell-autonomous transcriptional mechanism for enhancement of translation capacity in secretory cells [ChIP-Seq]
GSM3856429 FLAG ChIPseq in AtT-20 cells expressing 3x flag-Creb3l2 (cleaved form)
GSM3856430 XBP1 ChIPseq in AtT-20 cells
GSE132321 Cell-autonomous transcriptional mechanism for enhancement of translation capacity in secretory cells [RNA-Seq AtT-20 cells]
GSM3856431 RNAseq in AtT-20 expressing Neo (control) replicate 1
GSM3856432 RNAseq in AtT-20 expressing Neo (control) replicate 2
GSM3856433 RNAseq in AtT-20 expressing Neo (control) replicate 3
GSM3856434 RNAseq in AtT-20 expressing active (ie cleaved) Creb3l2 replicate 1
GSM3856435 RNAseq in AtT-20 expressing active (ie cleaved) Creb3l2 replicate 3
GSM3856436 RNAseq in AtT-20 expressing active (ie spliced) XBP1 (XBP1s) replicate 1
GSM3856437 RNAseq in AtT-20 expressing active (ie spliced) XBP1 (XBP1s) replicate 2
GSM3856438 RNAseq in AtT-20 expressing active (ie spliced) XBP1 (XBP1s) replicate 3
GSM3856439 RNAseq in AtT-20 expressing active Creb3l2 and XBP1 replicate 1
GSM3856440 RNAseq in AtT-20 expressing active Creb3l2 and XBP1 replicate 2
GSM3856441 RNAseq in AtT-20 expressing active Creb3l2 and XBP1 replicate 3
GSE132322 Cell-autonomous transcriptional mechanism for enhancement of translation capacity in secretory cells [RNA-Seq IL]
GSM3856442 RNAseq in WT IL replicate 1
GSM3856443 RNAseq in WT IL replicate 2
GSM3856444 RNAseq in POMC_ACreb3l2 Tg IL replicate 1
GSM3856445 RNAseq in POMC_ACreb3l2 Tg IL replicate 2
GSM3856446 RNAseq in POMC_AXBP1 Tg IL replicate 1
GSM3856447 RNAseq in POMC_AXBP1 Tg IL replicate 2
GSM3856448 RNAseq in POMC_ACreb3l2/AXBP1 double Tg IL replicate 1
GSM3856449 RNAseq in POMC_ACreb3l2/AXBP1 double Tg IL replicate 2

Genome browser session
(e.g. UCSC)

N/A

Methodology

Replicates

ChIPseq experiments were performed once.

Sequencing depth

ChIPseq Flag in AtT-20 cells expressing 3x Flag-Creb3l2 (cleaved form): 121 394 474 paired end reads PE50
XBP1 ChIPseq in AtT-20: 31 687 599 reads PE36
RNAseq in WT IL replicate 1: 66 769 763 paired end reads PE50
RNAseq in WT IL replicate 2: 69 907 906 paired end reads PE50
RNAseq in ACreb3l2 Tg IL replicate 1: 72 562 476 paired end reads PE50
RNAseq in ACreb3l2 Tg IL replicate 2: 62 985 370 paired end reads PE50
RNAseq in AXBP1 Tg IL replicate 1: 95 288 637 paired end reads PE50
RNAseq in AXBP1 Tg IL replicate 2: 75 671 798 paired end reads PE50
RNAseq in ACreb3l2/AXBP1 Tg IL replicate 1: 84 192 134 paired end reads PE50
RNAseq in ACreb3l2/AXBP1 Tg IL replicate 2: 76 013 144 paired end reads PE50
RNAseq in AtT-20 expressing Neo replicate 1: 24 183 212 paired end reads PE50
RNAseq in AtT-20 expressing Neo replicate 2: 30 945 186 paired end reads PE50
RNAseq in AtT-20 expressing Neo replicate 3: 33 464 937 paired end reads PE50
RNAseq in AtT-20 active (ie cleaved) Creb3l2 replicate 1: 25 667 290 paired end reads PE50
RNAseq in AtT-20 active (ie cleaved) Creb3l2 replicate 3: 33 153 072 paired end reads PE50
RNAseq in AtT-20 active (ie spliced) XBP1 (XBP1s) replicate 1: 33 377 398 paired end reads PE50
RNAseq in AtT-20 active (ie spliced) XBP1 (XBP1s) replicate 2: 31 314 664 paired end reads PE50
RNAseq in AtT-20 active (ie spliced) XBP1 (XBP1s) replicate 3: 37 222 030 paired end reads PE50
RNAseq in AtT-20 active Creb3l2 and XBP1 replicate 1: 31 885 110 paired end reads PE50
RNAseq in AtT-20 active Creb3l2 and XBP1 replicate 2: 27 260 477 paired end reads PE50
RNAseq in AtT-20 active Creb3l2 and XBP1 replicate 3: 52 409 384 paired end reads PE50

Antibodies

Flag: F3165 Sigma
XBP1: M-186, Santa Cruz Sc-7160
Creb3l2: NOVUS Biologicals NBP1-88697
rabbit IgG: Sigma G2018
mouse IgG: Sigma I5381

Peak calling parameters

Peaks were identified by comparing Creb3l2 to flag (GSM2324113) and XBP1 to IgG (GSM2324114) using MACS callpeak function with the parameters: --bw 250 -g mm --mfold 7,20 -p 1e-5 ; the parameter -f BAMPE was added for Creb3l2.

Data quality

Software	HOMER, Samtools, STAR 2.5, Trimmomatic 0.36, picard 2.17.3, MACS, Bowtie v.2.3.1, Deseq, Deseq2 (in Bioconductor), htseq-count, cufflinks 2.2.4, R_Bioconductor v3.5.1_3.7

Flow Cytometry

Plots

Confirm that:

- [x] The axis labels state the marker and fluorochrome used (e.g. CD4-FITC).
- [x] The axis scales are clearly visible. Include numbers along axes only for bottom left plot of group (a 'group' is an analysis of identical markers).
- [ ] All plots are contour plots with outliers or pseudocolor plots.
- [ ] A numerical value for number of cells or percentage (with statistics) is provided.

Methodology

Sample preparation	FACS analyses were performed at least 3 times on pools of 5-7 WT or KO adult mice pituitary intermediate lobe (IL) tissues. IL cells were dissociated as described in methods.
Instrument	FACSCalibur (BD) was used for FACS analyses
Software	Analysis of FACS data was performed using Summit
Cell population abundance	*Describe the abundance of the relevant cell populations within post-sort fractions, providing details on the purity of the samples and how it was determined.*
Gating strategy	FSC and SSC gates were set to avoid debris and doublets of cells. Propidium iodide labeling was used to exclude dead/dying cells.

- [x] Tick this box to confirm that a figure exemplifying the gating strategy is provided in the Supplementary Information.

Magnetic resonance imaging

Experimental design

Design type	*Indicate task or resting state; event-related or block design.*
Design specifications	*Specify the number of blocks, trials or experimental units per session and/or subject, and specify the length of each trial or block (if trials are blocked) and interval between trials.*
Behavioral performance measures	*State number and/or type of variables recorded (e.g. correct button press, response time) and what statistics were used to establish that the subjects were performing the task as expected (e.g. mean, range, and/or standard deviation across subjects).*

Acquisition

Imaging type(s)	*Specify: functional, structural, diffusion, perfusion.*
Field strength	*Specify in Tesla*
Sequence & imaging parameters	*Specify the pulse sequence type (gradient echo, spin echo, etc.), imaging type (EPI, spiral, etc.), field of view, matrix size, slice thickness, orientation and TE/TR/flip angle.*
Area of acquisition	*State whether a whole brain scan was used OR define the area of acquisition, describing how the region was determined.*

Diffusion MRI [ ] Used [ ] Not used

Preprocessing

Preprocessing software	*Provide detail on software version and revision number and on specific parameters (model/functions, brain extraction, segmentation, smoothing kernel size, etc.).*
Normalization	*If data were normalized/standardized, describe the approach(es): specify linear or non-linear and define image types used for transformation OR indicate that data were not normalized and explain rationale for lack of normalization.*
Normalization template	*Describe the template used for normalization/transformation, specifying subject space or group standardized space (e.g. original Talairach, MNI305, ICBM152) OR indicate that the data were not normalized.*
Noise and artifact removal	*Describe your procedure(s) for artifact and structured noise removal, specifying motion parameters, tissue signals and physiological signals (heart rate, respiration).*

Volume censoring | *Define your software and/or method and criteria for volume censoring, and state the extent of such censoring.*

Statistical modeling & inference

Model type and settings | *Specify type (mass univariate, multivariate, RSA, predictive, etc.) and describe essential details of the model at the first and second levels (e.g. fixed, random or mixed effects; drift or auto-correlation).*

Effect(s) tested | *Define precise effect in terms of the task or stimulus conditions instead of psychological concepts and indicate whether ANOVA or factorial designs were used.*

Specify type of analysis: ☐ Whole brain ☐ ROI-based ☐ Both

Statistic type for inference
(See Eklund et al. 2016) | *Specify voxel-wise or cluster-wise and report all relevant parameters for cluster-wise methods.*

Correction | *Describe the type of correction and how it is obtained for multiple comparisons (e.g. FWE, FDR, permutation or Monte Carlo).*

Models & analysis

n/a	Involved in the study
☐	☐ Functional and/or effective connectivity
☐	☐ Graph analysis
☐	☐ Multivariate modeling or predictive analysis

Functional and/or effective connectivity | *Report the measures of dependence used and the model details (e.g. Pearson correlation, partial correlation, mutual information).*

Graph analysis | *Report the dependent variable and connectivity measure, specifying weighted graph or binarized graph, subject- or group-level, and the global and/or node summaries used (e.g. clustering coefficient, efficiency, etc.).*

Multivariate modeling and predictive analysis | *Specify independent variables, features extraction and dimension reduction, model, training and evaluation metrics.*
